# Physical-Field Engineering of Food Hydrogels: Multiscale Assembly, Processing Windows, and Functional Translation

**DOI:** 10.3390/gels12100879

**Published:** 2026-09-28

**Authors:** Jialong Xu, Sulafa B. H. Hashim, Haoyang Xu, Aocheng Zhang, Jun Ren, Xiaobo Zou, Qing Sun, Jiyong Shi

**Affiliations:** 1 School of Food Science and Engineering, Jiangsu University, Zhenjiang 212013, China; 2 Department of Food Technology, Faculty of Agricultural Technology and Fish Sciences, Alneelain University, P.O. Box 12702, Khartoum 11121, Sudan; 3 Meihua Catering Management Co., Ltd., Nanjing 210006, China

**Keywords:** food hydrogels, physical fields, field-mediated gelation, energy deposition, multiscale network assembly, structure–function relationships

## Abstract

Food hydrogels are multiscale, water-rich networks whose functionality depends not only on composition but also on how processing energy is deposited and redistributed during network formation. Although acoustic, electric, magnetic, and pressure-based treatments have expanded the available strategies for hydrogel structuring, current evidence remains fragmented by inconsistent dose reporting, thermal and electrochemical confounding, strong matrix dependence, limited validation in compositionally realistic foods, and insufficient scale-up and safety assessment. This review critically evaluates these technologies through a field–process–structure–function framework that links measurable field inputs and energy deposition to molecular and colloidal reconfiguration, network architecture, water mobility, and food functionality. Across the available literature, ultrasound has the broadest evidence base for precursor restructuring and gel network modification, while high-pressure processing shows comparatively strong support for direct gelation and composite network reinforcement. Electric-field approaches offer controllable electrothermal and directional effects but require more rigorous separation of field-specific contributions from thermal and electrochemical effects. Magnetic and conductive edible hydrogels remain comparatively early-stage, with much of the mechanistic evidence still derived from non-ingestible systems. Overall, beneficial responses occur within matrix-specific processing windows rather than increasing monotonically with treatment intensity. Future progress requires matched controls, quantitative dose reporting, realistic food matrices, food-grade safety validation, continuous-processing equipment, and energy-normalized assessment of technological feasibility.

## 1. Introduction

Hydrogels are three-dimensional networks of hydrophilic macromolecules that can immobilize large quantities of water while maintaining a solid-like response over relevant timescales [1]. In food systems, these networks are formed primarily from proteins, polysaccharides, starches, fibrils, particles, and combinations thereof. Their functions extend far beyond thickening. Hydrogel networks govern fracture behavior, lubrication, moisture retention, molecular diffusion, flavor release, disassembly during digestion, freeze–thaw stability, and shape fidelity during additive manufacturing (Figure 1A,B). Accordingly, recent reviews have shifted from material inventories toward structure–function analysis and theoretical descriptions of network mechanics [2,3]. This shift is important because gels with similar small-deformation moduli can exhibit markedly different pore architectures, fracture modes, water distributions, and oral-processing behaviors. Composition alone is, therefore, insufficient to describe food hydrogel performance.

Recent reviews have advanced the understanding of food hydrogels from several complementary perspectives. Emerging fabrication technologies have been summarized in terms of hydrogel sources, processing mechanisms, and applications, with ultrasound, high-pressure processing, moderate electric fields, and other technologies treated primarily as alternative fabrication routes [1]. Other reviews have focused on multilevel structural design across hydrogels, oleogels, and biphasic gels and their translation into food applications [2], or have examined protein- and polysaccharide-based hydrogels from a rheological perspective, emphasizing crosslinking strategies and structure–property relationships [3]. More recently, the mechanics, experimental characterization, theoretical description, and formulation of food hydrogels have been critically reviewed [4], while stimulus-responsive hydrogels have been organized according to their environmental triggers and potential food applications [5]. Collectively, these studies establish an important foundation for hydrogel formulation, characterization, and application, but they do not provide a common basis for comparing how distinct externally applied physical fields deposit energy, redirect network assembly, and generate beneficial or detrimental responses across different food matrices.

In this review, “physical-field engineering” is used operationally to describe processing in which an externally imposed acoustic, electric, magnetic, or hydrostatic pressure field acts as a primary controllable input and modifies molecular, colloidal, or network assembly through measurable force or energy transfer. The defining criterion is, therefore, not the absence of chemicals or heat, but the presence of a spatially and temporally controllable physical field whose interaction with the matrix can be characterized independently of subsequent thermal, mechanical, or chemical consequences. Technologies dominated primarily by bulk flow, shear, impact, or mechanical comminution are not treated as equivalent field modalities, even when they generate secondary cavitation or pressure fluctuations. Accordingly, high-pressure homogenization is included only as a related mechanical pretreatment for comparison with pressure-assisted precursor modification, whereas isostatic high-pressure processing is retained within the core framework because hydrostatic pressure itself constitutes the principal imposed physical variable.

The emerging concept of physical-field engineering incorporates processing history into this structure–function perspective. Acoustic, electric, magnetic, and pressure fields do not act on abstract polymer networks; rather, they transfer and dissipate energy within food matrices that are heterogeneous, conductive, compressible, and frequently multiphase. The local response is governed by protein source, polysaccharide charge, ionic strength, pH, solids loading, the presence of dispersed oil or particles, viscosity, thermal conductivity, and equipment geometry [6,7]. The same field may reduce particle size in one system while intensifying aggregation in another. A field may also accelerate gelation yet produce a weaker network or increase mechanical strength at the expense of water-holding capacity. These seemingly contradictory outcomes arise because molecular unfolding, intermolecular association, phase separation, transport processes, and irreversible damage proceed concurrently but at different rates [8].

Against this background, the principal knowledge gap is not the absence of information on individual physical-field technologies, but the lack of a common causal framework for judging when and why a field-mediated treatment produces a distinct hydrogel structure or function. This review, therefore, conceptualizes physical fields as spatiotemporal regulators of network assembly rather than simply as emerging or nonthermal processing technologies. The proposed field–process–structure–function framework differs from technology- or material-centered classifications by explicitly separating the nominal field input from the energy actually deposited in the matrix and by tracing this input through molecular and colloidal reconfiguration, hydrated network architecture, and food-level functionality. It further distinguishes structural priming before gelation, field-mediated intervention during network formation, and post-gel actuation or restructuring, because these treatment modes involve different causal pathways. A further emphasis is placed on matrix-dependent processing windows, including both beneficial responses and failure modes, and on the strength of evidence required to separate field-specific effects from thermal, mechanical, or electrochemical contributions. By integrating these mechanistic criteria with quantitative treatment conditions, safety, energy demand, scale-up, and functional translation, the framework provides a basis for comparing otherwise non-equivalent physical-field approaches and for identifying where current evidence remains insufficient for food applications. For evidential clarity, the studies considered here are classified as direct food hydrogel applications, food contact applications, or mechanistic inspiration from non-food systems; only the first category is treated as direct evidence for edible food hydrogel structuring and functionality.

### 1.1. Food Hydrogels Behave as Dynamic Multiscale Systems

A food hydrogel is best regarded as a dynamic, hierarchical system. At the molecular scale, hydrogen bonding, electrostatic attraction and repulsion, hydrophobic association, disulfide exchange, coordination interactions, and covalent crosslinking determine junction lifetime and reversibility. At the colloidal scale, soluble aggregates, insoluble particles, fibrils, droplets, and polysaccharide-rich domains function as load-bearing elements or stress concentrators [9]. At the mesoscale, these elements assemble into homogeneous, particulate, bicontinuous, phase-separated, filled, interpenetrating, or double networks. Water permeates and couples all levels of this hierarchy. It plasticizes polymer chains, mediates stress transfer, regulates molecular mobility, enables diffusion, and determines whether the network remains cohesive or undergoes syneresis [10].

The dynamic character of hydrogel networks becomes evident under heating, acidification, salt addition, shear, storage, digestion, and repeated deformation. Weak physical junctions can dissociate and reform, thereby enabling thixotropic recovery and supporting printability (Figure 1C). Covalently crosslinked or densely associated domains can increase stiffness but may restrict extensibility and molecular transport. Phase separation can be beneficial when it generates a hierarchical load-bearing architecture, but detrimental when it produces coarse interfaces that promote fracture or water expulsion. Insoluble particles can reinforce a sparse matrix below the particle percolation threshold, but act as fracture initiators when they form large, poorly bonded inclusions [11]. Field engineering must, therefore, target the balance among junction density, network connectivity, structural heterogeneity, water binding, and damage tolerance rather than any single structural variable.

### 1.2. Conventional Processing Provides Limited Control over Food Hydrogel Assembly

Heat-set gelation is robust and industrially established, but it often couples the desired unfolding of proteins with poorly controlled aggregation. Temperature gradients can become pronounced in highly viscous or large-format products, while prolonged holding may promote flavor deterioration, nutrient degradation, surface-skin formation, and water redistribution. Cold-set gelation decouples protein unfolding from subsequent network formation but generally requires prior thermal treatment followed by the addition of salts, acids, or enzymes. Ionotropic polysaccharide gelation is rapid but can generate steep crosslink density gradients as ions diffuse inward from the product surface. Enzymatic routes offer reaction specificity, although reaction kinetics and the spatial distribution of the enzyme remain sensitive to temperature, substrate accessibility, and matrix composition [12].

These limitations do not imply that physical fields should universally replace chemical, ionic, or enzymatic crosslinking. Conventional crosslinking routes remain advantageous when robust gelation can be achieved through well-defined composition and reaction chemistry, particularly when spatial or temporal control of network formation is not required. Their controllability, however, is often constrained by reagent distribution, diffusion, reaction kinetics, and the accessibility of reactive sites within heterogeneous food matrices. Physical fields provide an additional process control dimension because field strength, waveform, treatment duration, spatial distribution, and treatment sequence can be adjusted during processing. This enables precursor priming, volumetric energy delivery, directional transport or alignment, and localized activation of network formation without necessarily increasing the concentration of reactive crosslinkers. Physical-field processing is, therefore, most justified when dynamic control over where, when, or how the network assembles provides a functional advantage that cannot be achieved efficiently by composition control alone. These potential benefits must still be balanced against energy demand, equipment complexity, safety, reproducibility, and scale-up constraints [4,13,14].

**Figure 1:**
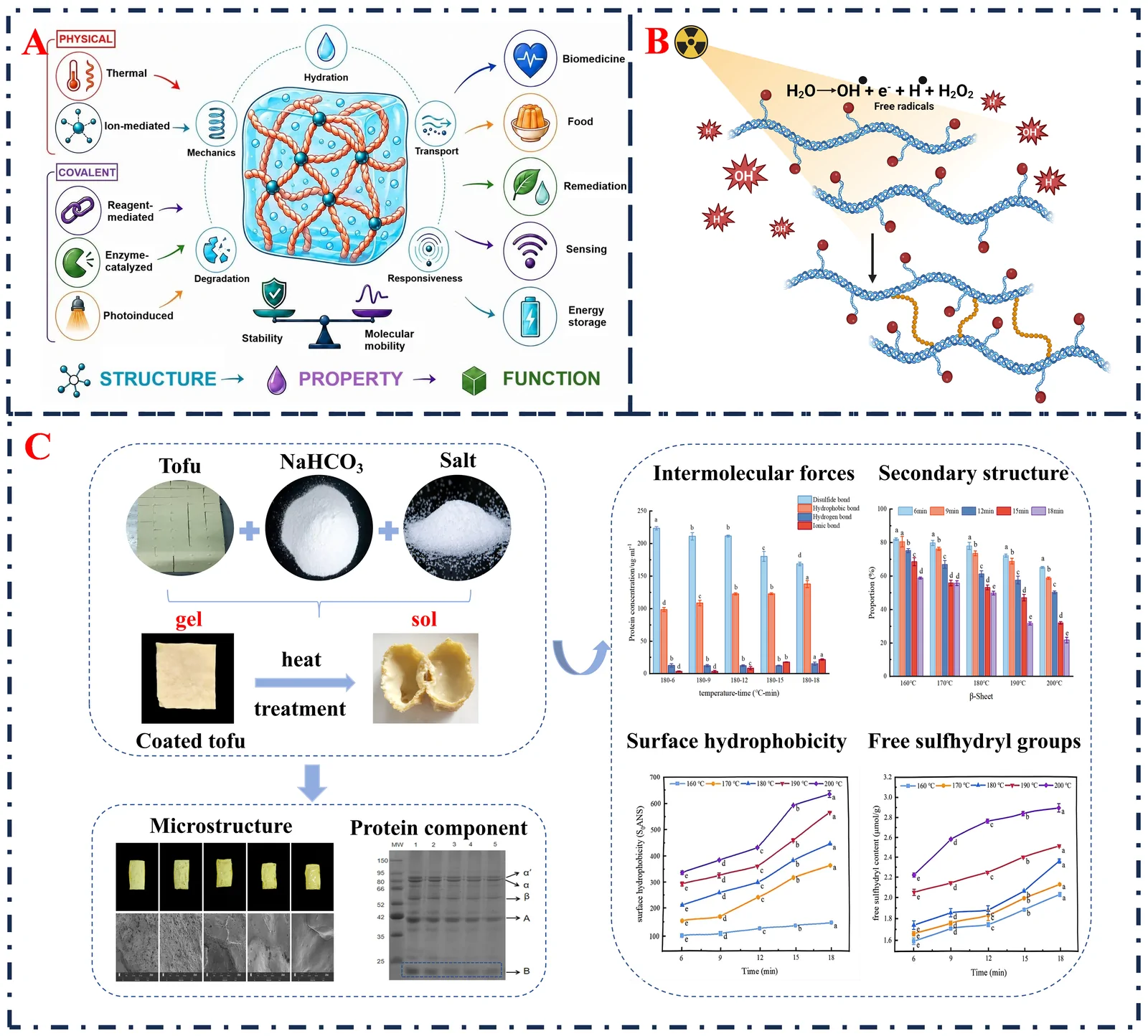
Representative examples illustrating how hydrogel network formation links crosslinking mechanism, processing history, and functionality. (**A**) Gelatin hydrogel design illustrates how physical and covalent junctions regulate network architecture and downstream material functions [15]. (**B**) Radiation-induced radical crosslinking illustrates an alternative route to three-dimensional network formation and highlights the dependence of hydrogel architecture on crosslinking mechanism [16]. (**C**) Heat-induced gel–sol transition in coated tofu demonstrates how thermal processing can reorganize protein structure and destabilize a food gel network [17].

### 1.3. Physical Fields Regulate Hydrogel Assembly Across Space and Time

Physical fields differ in the pathways through which energy or force is transferred to a food matrix. Acoustic systems couple through pressure-wave propagation and cavitation (Figure 2A); electric systems through charge transport, polarization, and Joule heating; magnetic systems primarily through responsive inclusions (Figure 2B); and high-pressure processing through isostatic compression (Figure 2C). Their common engineering advantage lies in the ability to regulate treatment intensity, spatial distribution, temporal sequence, and exposure history. However, nominal instrument settings do not represent the local dose experienced by heterogeneous food matrices. Mechanistic comparison, therefore, requires physically meaningful descriptors of energy deposition, temperature history, field distribution, conductivity, particle organization, or pressure–time trajectories rather than nominal power or field strength alone [18,19].

## 2. Physical Fields Regulate Hydrogel Assembly Through Multiscale Energy Transfer and Matrix Responses

### 2.1. Applied Fields Deliver and Redistribute Energy Through Distinct Transport Pathways

The response of a hydrogel precursor can be conceptualized as the coupling between an applied field and the matrix-specific susceptibility of the material. Acoustic susceptibility depends on density, compressibility, viscosity, dissolved-gas content, interfacial tension, and the presence and distribution of particles or bubbles (Figure 3A) [23]. Electrical susceptibility is governed by conductivity, permittivity, field frequency, ionic mobility, and interfacial electrode reactions (Figure 3B) [24]. Magnetic susceptibility is intrinsically low in most native food biopolymers; effective magnetic coupling, therefore, generally requires responsive inclusions or an indirect transduction mechanism (Figure 3C) [25]. Pressure susceptibility is governed by partial molar volume, compressibility, hydration, and the volume changes accompanying molecular dissociation, unfolding, aggregation, and phase transitions. Heat and mass transfer subsequently redistribute locally deposited energy and determine whether the initial field-induced response persists, evolves, or dissipates [26].

#### 2.1.1. Acoustic Cavitation Dissipates Mechanical Energy Through Bubble Oscillation and Collapse

Low-frequency, high-intensity ultrasound, as commonly applied in food processing, generates alternating compression and rarefaction cycles. Pre-existing gas nuclei expand and may undergo stable oscillation or transient collapse. Cavitation bubble collapse near a solid or droplet interface generates microjets and steep velocity gradients, whereas collapse in the bulk induces localized heating, shock waves, turbulence, and radical formation. These events can disrupt weakly associated protein particles, reduce the apparent size of aggregates, expose buried hydrophobic or sulfhydryl groups, accelerate hydration, and modify macromolecular collision frequencies [27,28]. Cavitation intensity is progressively attenuated with increasing viscosity and solids content. Consequently, a treatment that is effective in a dilute protein dispersion may generate a substantially different cavitation environment in a concentrated pre-gel system or emulsion.

Mechanical and thermal contributions cannot be distinguished unless experiments are explicitly designed to decouple them. Recent work on soy protein has shown that ultrasound-induced heat accumulation can contribute directly to protein unfolding and subsequent network crosslinking [29]. A conventionally heated, matched-temperature control is necessary but may remain insufficient because heating rates and local temperature distributions can differ between treatments. Chemical dosimetry, calorimetric power measurements, passive cavitation detection, and hydrophone measurements provide complementary evidence regarding chemical, thermal, and acoustic energy deposition. Reporting only amplitude or nominal process setting, without specifying vessel volume, probe diameter, immersion depth, pulse mode, and temperature-control conditions, is insufficient for mechanistic interpretation or cross-study comparison.

#### 2.1.2. Electric Fields Drive Electrophoretic Transport and Dielectric Polarization

Direct-current and low-frequency electric fields can drive the migration of charged proteins, polysaccharides, ions, and particles toward oppositely charged electrodes. This migration can generate concentration gradients and promote the orientation of elongated aggregates or polymer chains. Alternating electric fields limit net electrophoretic displacement but can induce dipolar polarization and polarization of interfacial charge layers. During ohmic heating, electrical current passing through the sample generates volumetric Joule heating, which can reduce macroscopic temperature gradients relative to surface-mediated heating [30]. Moderate electric fields may also influence aggregation and gelation through mechanisms not fully explained by the imposed thermal history, although the magnitude and direction of these effects depend on protein source, field frequency and strength, sample conductivity, and electrode configuration (Figure 3D) [31,32].

Electrochemical reactions at the electrode–sample interface can confound the mechanistic interpretation of electric-field treatments. Local pH shifts, gas evolution, metal-ion release, and the formation of redox-active products near the electrodes may promote, redirect, or inhibit gelation. These effects are not inherently undesirable; for example, electrochemical acidification can be deliberately exploited as a gelation trigger. Nevertheless, these contributions must be quantified and distinguished from effects attributed to field-induced alignment or polarization. Electrode material, exposed surface area, interelectrode spacing, current density, polarity reversal protocol, and sample conductivity should, therefore, be treated as primary process variables rather than ancillary equipment specifications [33].

**Figure 3:**
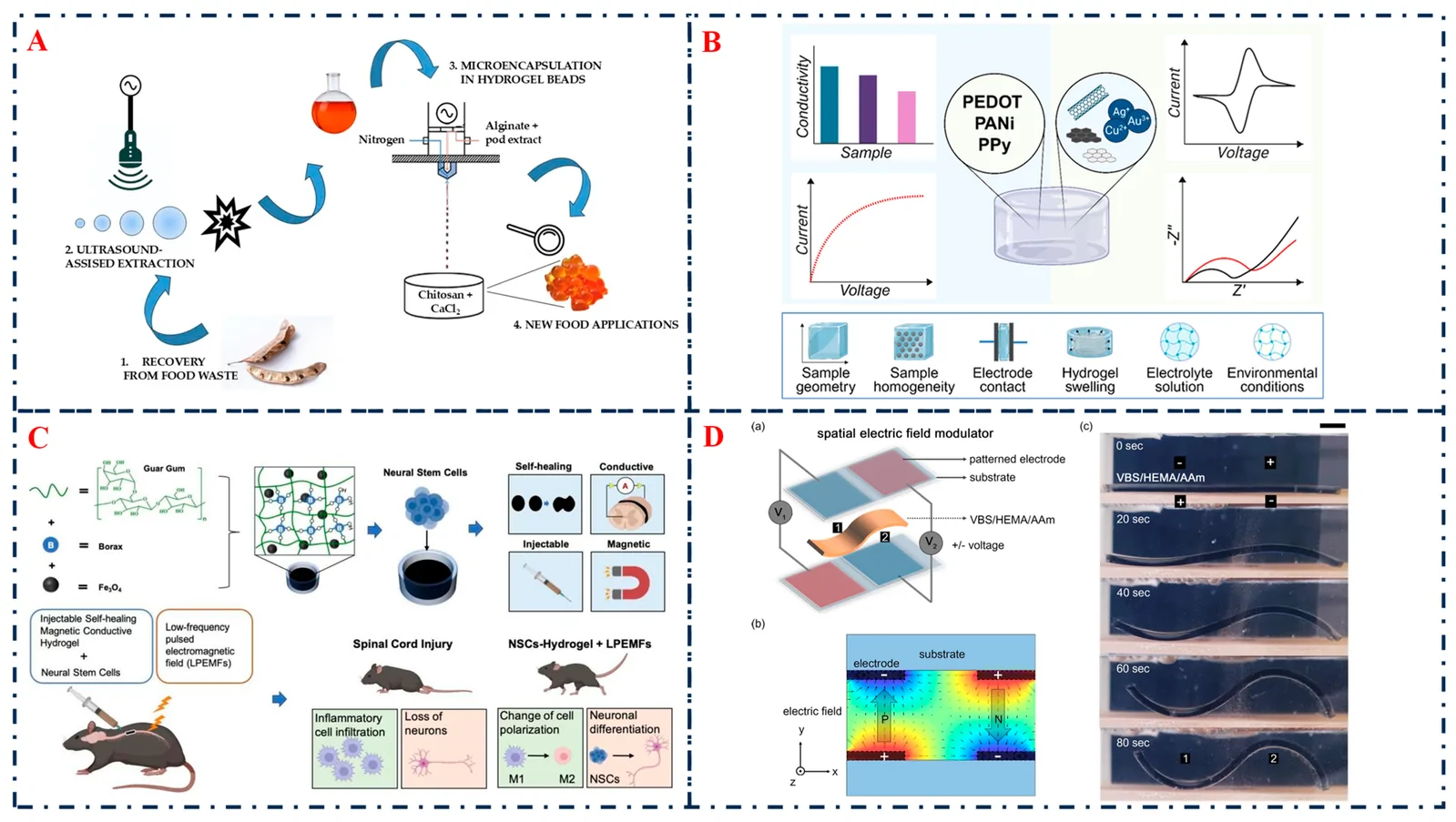
Representative physical-field coupling mechanisms shown across different evidence contexts. (**A**) Ultrasound-assisted processing followed by hydrogel encapsulation represents a food-related application of acoustic-field processing [34]. (**B**) Electrical characterization of conductive hydrogels is presented as mechanistic inspiration from a biomedical system to illustrate ionic and electronic transport behavior [24]. (**C**) A low-frequency pulsed electromagnetic-field-responsive biomedical hydrogel is included as mechanistic inspiration for remote field coupling and responsive network design [35]. (**D**) Electric-field-induced hydrogel morphing provides mechanistic inspiration for spatially controlled electroactive deformation [36].

#### 2.1.3. Magnetic Fields Generate Forces, Torques, and Magnetorheological Responses

Most food proteins and polysaccharides exhibit only weak intrinsic magnetic susceptibility. Effective magnetic structuring, therefore, generally relies on dispersed iron oxides, magnetically responsive fibers, or other inclusions that convert field gradients into translational forces and magnetic moments into rotational torque. These inclusions may align, assemble into chains, compact local polymer domains, or generate heat when exposed to alternating magnetic fields. Subsequent network formation can immobilize and preserve the field-induced organization. A recent study reported that low-frequency magnetic field treatment enhanced gelation and structural organization in potato protein emulsion gels, providing preliminary evidence for direct application in food hydrogel structuring [37]. Magnetic regulation has also been integrated with Fe_3_O_4_-containing, chitosan-based hydrogel coatings to support photothermal antimicrobial preservation of beef [38].

The mechanistic evidence for magnetic-field-mediated structuring remains considerably less developed than that available for ultrasound or high-pressure processing. Magnetic particles can alter local ionic conditions, surface chemistry, optical absorption, and nucleation behavior even under field-free conditions [39]. Alternating magnetic fields introduce a magnetothermal pathway, whereas permanent magnets produce spatially heterogeneous field gradients that may decay sharply with distance. Studies should, therefore, include particle-free controls, field-free particle-containing controls, matched-temperature controls, quantitative measurements of particle migration or alignment, and migration testing under intended food-contact conditions. Magnetic responsiveness alone should not be interpreted as evidence of edibility, food-contact safety, or regulatory acceptability.

#### 2.1.4. Hydrostatic Pressure Shifts Volume-Dependent Equilibria and Molecular Interactions

High-pressure processing applies hydrostatic pressures, commonly in the range of hundreds of megapascals, throughout packaged or otherwise contained samples. Pressure shifts physicochemical equilibria according to the volume change associated with each transition. Pressure-induced dissociation of multimeric proteins, changes in hydration, partial unfolding, and subsequent reassembly may occur without the prolonged thermal history required for conventional heat-induced gelation. Covalent bonds are relatively resistant to pressure, whereas hydrophobic interactions, electrostatic interactions, and other noncovalent forces can be substantially perturbed. The resulting gels may exhibit fine-stranded, particulate, homogeneous, or phase-separated architectures, depending on pH, protein concentration, pressure level, holding time, treatment temperature, and the presence of co-solutes [40,41].

High-pressure homogenization (HPH) is discussed here only as a related mechanical pretreatment rather than as an equivalent physical-field modality. Unlike isostatic HPP, HPH subjects a flowing dispersion to intense shear, turbulence, impact, and cavitation while the material passes through a narrow valve or interaction chamber. These stresses can reduce particle size, modify interfacial properties, and alter subsequent acid- or heat-induced gelation, but the imposed valve pressure is not transmitted uniformly through the sample as a hydrostatic field. For example, HPH pretreatment has been shown to modify sesame protein gelation by reducing particle size and promoting a denser network, with the most favorable response occurring at an intermediate homogenization pressure [42,43]. HPH is, therefore, retained only as a mechanistic comparator for pressure-related precursor modification and is excluded from the core physical-field classification used throughout this review.

#### 2.1.5. Coupled Heat, Momentum, and Mass Transfer Determine Field-Mediated Gelation

Field-mediated gelation is ultimately governed by coupled heat, momentum, and mass-transfer processes. Ultrasound modifies bulk mixing and interfacial renewal; electric fields alter ionic transport and can generate volumetric heating; magnetic particles may migrate or produce localized heating; and pressure modifies diffusion, phase equilibria, and protein hydration. These transport processes proceed as viscosity increases and molecular or particulate mobility progressively decreases. Transport during the early stages of gelation can homogenize the precursor, whereas transport occurring near or after network percolation may preserve compositional or structural gradients within the forming gel [44]. The timing of field application relative to the sol–gel transition may, therefore, be as consequential as the nominal treatment dose.

A practical implication is that precursor composition, rheology, and reactor geometry must be considered jointly during process design. Unlike purified protein or polysaccharide dispersions, realistic food matrices contain salts, sugars, lipids, and dispersed particles that modify both field coupling and the intrinsic gelation pathway. In electric-field processing, dissolved salts increase ionic conductivity and can enhance Joule heating at a given field strength, whereas spatial variations in salt concentration may redistribute current density and generate nonuniform energy deposition. Sugars modify water mobility, viscosity, ionic mobility, and dielectric properties in a concentration-, temperature-, and frequency-dependent manner, thereby altering electrical energy dissipation. Lipid phases generally exhibit much lower permittivity and conductivity than water-rich continuous phases; dispersed lipid droplets can, therefore, introduce local dielectric heterogeneity and modify field distribution. Composition also governs acoustic propagation. Acoustic impedance depends on material density and sound velocity, both of which vary with solids composition and temperature, while oil–water interfaces introduce impedance contrasts that promote reflection and scattering. Increasing sugar or biopolymer concentration can additionally increase viscosity and acoustic attenuation, thereby suppressing cavitation and reducing the effective treatment volume. Salts, sugars, and lipids may simultaneously alter protein hydration, electrostatic screening, interfacial interactions, and network formation. Consequently, treatment conditions optimized in simplified biopolymer solutions cannot be transferred directly to compositionally complex foods without characterizing conductivity or dielectric behavior, acoustic propagation, temperature history, and matrix rheology under the intended formulation conditions.

### 2.2. Molecular Unfolding, Association, and Crosslinking Jointly Determine Network Formation

Protein unfolding is frequently invoked to explain field-enhanced gelation, yet greater unfolding does not necessarily produce a mechanically stronger network. An effective precursor must expose reactive interaction sites while remaining sufficiently dispersed to participate in the formation of a continuous network. Excessive unfolding can promote the formation of insoluble aggregates before gelation, thereby depleting available network-forming species and introducing structural defects that initiate fracture. At moderate treatment intensities, ultrasound and electric fields may reduce aggregate size and increase surface hydrophobicity, whereas higher doses can promote reaggregation. Pressure may dissociate oligomeric structures and expose hydrophobic regions, followed by pressure-history-dependent reassembly upon decompression [45,46]. The decisive factor is, therefore, the transition pathway from the native state through soluble aggregates to a percolated network, rather than the magnitude of any single spectroscopic response.

Polysaccharides may respond through chain scission or disentanglement, changes in hydration and alignment, ion binding, and spatial redistribution [45]. In mixed protein–polysaccharide systems, an applied field can modify both biopolymer components and the relative rates at which they restructure and associate. Rapid protein unfolding and association may precede polysaccharide network formation, thereby producing a composite with a protein continuous phase. Conversely, rapid polysaccharide gelation can immobilize protein aggregates within the network as dispersed fillers. Electrostatic complexation may reinforce network connectivity or, under unfavorable charge and compositional conditions, promote macroscopic phase separation. Mechanistic assignments require targeted inhibition or perturbation experiments, such as the selective disruption of hydrogen bonding, hydrophobic interactions, disulfide bonds, or ionic bridges, combined with rheological and microstructural characterization. Spectral peak shifts alone cannot uniquely identify the interactions responsible for network formation [47].

### 2.3. Mesoscale Organization Connects Molecular Responses with Anisotropy and Porosity

Mesoscale organization provides the structural link between molecular-level modification and macroscopic functionality. Physical fields can modify aggregate aspect ratio, particle surface roughness, droplet-size distribution, interfacial protein coverage, pore connectivity, and the dimensions of phase-separated domains (Figure 4A). A visually denser micrograph does not necessarily indicate a mechanically stronger or more stable network; sample drying during electron microscopy can collapse pores, and a finely structured network may remain brittle if its junctions cannot dissipate or redistribute applied stress. Confocal microscopy, cryogenic imaging, X-ray tomography, scattering methods, particle tracking, and nonlinear rheology can provide complementary information on hydrated network architecture and deformation under load [48].

Structural anisotropy is a particularly promising outcome of field-directed assembly. Low-voltage electric fields have been used to produce oriented soy protein hydrogels and soy protein/konjac-glucomannan double networks under high-moisture conditions [49]. Directional organization can generate distinct longitudinal and transverse mechanical responses, potentially enhancing fibrous perception, directional transport, or field-responsive actuation. Evidence of field-induced anisotropy should include quantitative orientation metrics, direction-dependent mechanical testing, and assessment of structural persistence after removal of the field. Visual evidence of alignment alone is insufficient to establish functionally meaningful anisotropy.

### 2.4. Matrix Composition and Processing History Determine Susceptibility to Physical Fields

Protein origin is a major determinant of field susceptibility because globulin composition, sulfur-containing amino acid content, native oligomeric state, extraction-induced structural damage, and insoluble particle content vary substantially among ingredients. Commercial plant protein isolates may contain substantial insoluble fractions that respond differently to ultrasound or pressure compared with more highly purified laboratory-prepared isolates (Figure 4B) [11]. Polysaccharide charge density and chain conformation influence whether the polysaccharide forms complexes with proteins, partitions into a separate phase, or participates in an interpenetrating network. pH determines protein net charge and proximity to the isoelectric point, whereas ionic strength governs electrostatic screening and contributes to the electrical conductivity of the matrix. Solids content influences intermolecular and interparticle collision frequencies, bulk viscosity, acoustic attenuation, and the likelihood of network percolation [12].

Susceptibility to field treatment also depends on the processing history of the ingredient. Spray drying, alkaline extraction, oxidation, freeze–thaw cycling, and prior heating can alter the conformational states and intermolecular interactions accessible during subsequent processing. When combined with pH shifting, ultrasound may partially restore the gelation capacity of oxidized or poorly soluble proteins, whereas the same treatment may induce excessive aggregation in more conformationally labile proteins (Figure 4C) [50]. Field parameters should, therefore, be optimized for well-characterized ingredient lots rather than transferred among materials solely on the basis of their nominal protein source.

Cross-study differences should, therefore, be interpreted in relation to matrix state rather than nominal field setting alone. As summarized in Table 1, the same class of treatment can generate substantially different outcomes when protein source, subunit composition, ionic strength, polysaccharide content, or prior processing changes. In low-voltage electric-field systems, increasing ionic strength can strengthen the gel while simultaneously reducing water-holding stability, whereas incorporation of κ-carrageenan markedly reinforces pressure-induced pea protein networks. Ultrasound responses likewise vary with extraction history and protein subunit composition, while ohmic-heating responses differ among pea, potato, and egg white proteins. These contrasts indicate that field–structure–function relationships emerge from the interaction among energy deposition, molecular susceptibility, aggregation kinetics, network percolation, and water redistribution. Quantitative treatment conditions should, therefore, be interpreted as material-specific processing windows rather than transferable technology-wide optima [32,41].

**Figure 4:**
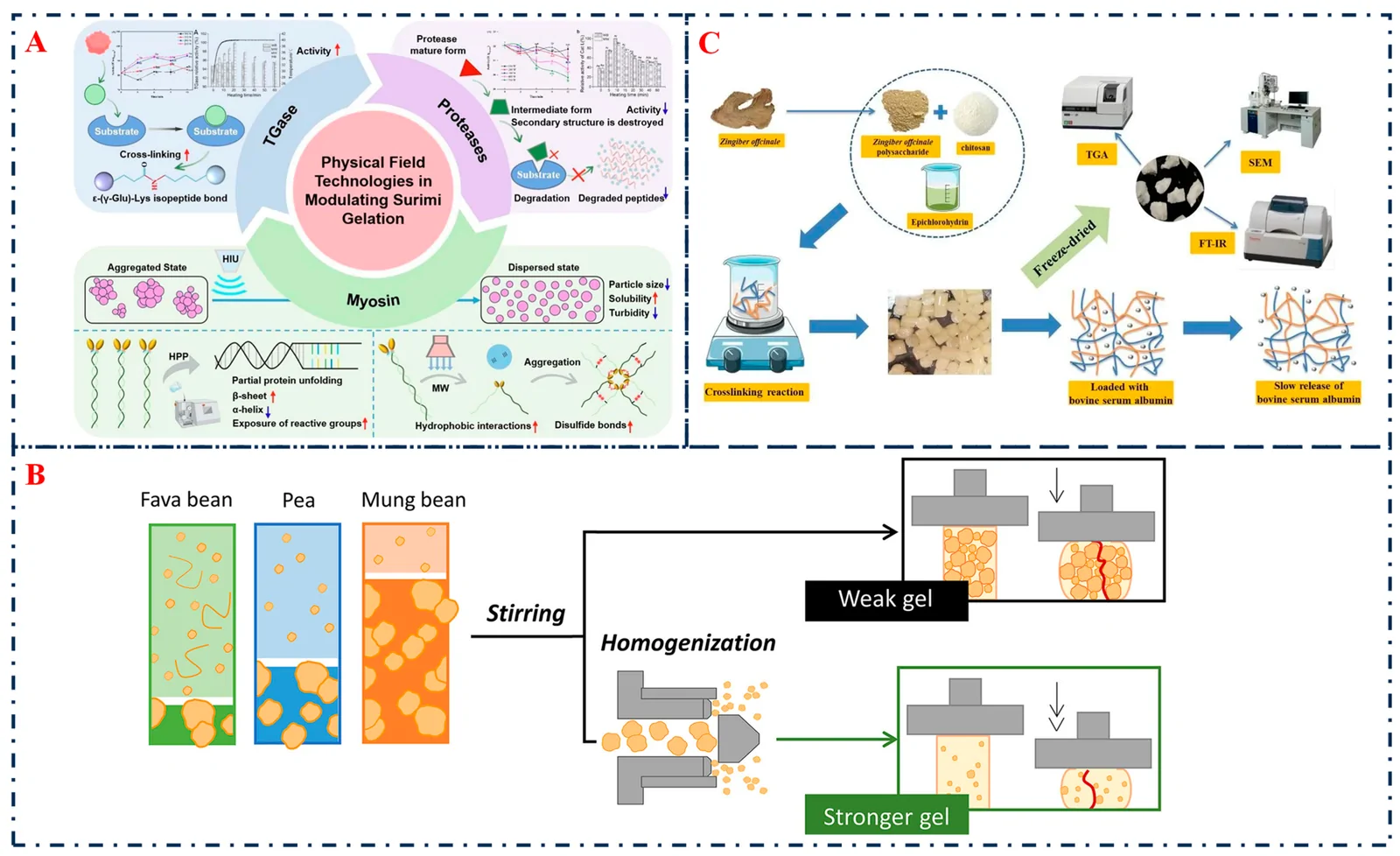
Representative examples illustrating how field treatment and matrix characteristics jointly regulate hydrogel organization and functionality. (**A**) Physical-field regulation of surimi gelation illustrates how different energy inputs can alter protein assembly and mesoscale network development [51]. (**B**) Commercial plant protein isolates illustrate how insoluble particles and ingredient heterogeneity modify gelation behavior and matrix susceptibility [11]. (**C**) A chitosan–polysaccharide composite hydrogel illustrates how matrix composition and network architecture regulate structural organization and molecular release [52].

### 2.5. Competing Mechanisms Create Matrix-Dependent Processing Windows and Structural Failure Modes

Most relationships between field intensity and hydrogel response are nonmonotonic and matrix-dependent. At low treatment doses, energy deposition may be insufficient to modify dispersion or expose a functionally relevant number of reactive sites. Within an intermediate processing window, molecular mobility and collision frequency may increase in ways that promote network connectivity. Beyond this window, chain scission, oxidative modification, poorly controlled aggregation, excessive densification, phase separation, or water expulsion may outweigh the beneficial restructuring mechanisms. The optimum processing window, therefore, shifts with matrix composition and the functional attribute being prioritized. Conditions that maximize hardness may not maximize water retention, toughness, digestibility, or printability and may even compromise one or more of these attributes [5].

Structural failure modes should be treated as informative mechanistic outcomes rather than dismissed as undesirable or negative results. Ultrasound may generate radicals and accumulate heat; electric-field processing may produce electrode-derived species and local pH gradients; magnetic particles may aggregate or migrate; and high-pressure treatment may over-densify the network or promote aggregation after decompression. Mapping the transition from reversible restructuring to irreversible damage is more informative than reporting a single empirically optimized treatment condition. Factorial designs and response surface methods are useful, but mechanistically relevant descriptors—including absorbed energy, temperature history, conductivity, particle-size distribution, water mobility, and interaction strength—should be evaluated alongside empirical structural and quality responses [5,53]. Representative quantitative processing windows, including reported beneficial responses and experimentally observed upper-window limitations, are summarized in Table 1.

### 2.6. A Unified Framework Connects Physical-Field Inputs with Hydrogel Structure, Functionality, and Translational Feasibility

Physical-field engineering of food hydrogels is best represented as a continuous causal pathway in which measurable field inputs interact with matrix-specific susceptibility and reactor geometry to establish local energy deposition and transport. The applied field is defined by frequency, amplitude or field strength, waveform, pressure level, field gradient, duty cycle, and treatment duration, while equipment and sample geometry determine the spatial distribution of these inputs [5,6]. Their mechanistic significance depends on the resulting calorimetric or absorbed power, temperature history, electrical current and conductivity, cavitation activity, magnetic-particle displacement, and pressure–time trajectory. These local thermal, mechanical, electrical, magnetic, and pressure histories alter molecular and colloidal states through conformational unfolding, oligomer dissociation, aggregate disruption or reformation, interfacial rearrangement, and chemical modification. The extent to which these responses persist during gelation determines network connectivity, phase organization, structural anisotropy, pore architecture, and water mobility. The resulting hydrated architecture governs texture, fracture behavior, water-holding capacity, physical and storage stability, controlled release, digestibility, and performance in 3D food printing. Application-level benefits must then be evaluated against energy demand, throughput, process reproducibility, safety, cost, and consumer acceptance because structural modification alone does not establish technological value [6,46].

Mechanistic confidence increases when measured field conditions and energy deposition are linked to molecular or colloidal changes, hydrated network architecture, and application-level performance. Property changes alone remain associative unless relevant thermal, mechanical, electrochemical, or particle-mediated contributions are adequately controlled [54]. Figure 5 summarizes this evidence hierarchy, while Table 2 compares the major mechanistic and translational descriptors.

**Table 1:** Representative quantitative processing windows, structural and functional outcomes, and reported limitations of physical-field-engineered food hydrogels.

Field Modality	Hydrogel Matrix	Key Operating Conditions	Gelation/Intervention Route	Beneficial Structural or Functional Outcomes	Deterioration/Limitation	Reference
High-intensity ultrasound (HIU)	Soy protein isolate (SPI)	20 kHz; 400 W nominal power; 5, 10, 15, or 20 min; with or without ice-water temperature control; power density NR	Ultrasound pretreatment followed by GDL-induced gelation	Without temperature control, D [3,4] reached 20.53 ± 2.97 μm after 20 min; maximum gel strength reached 66.88 ± 7.07 g after 15 min	Heat accumulation contributed substantially to protein unfolding and gel strengthening; nominal acoustic power alone cannot distinguish thermal from cavitation effects	[29]
Low-voltage direct-current electric field	Soy protein isolate	1.5 V cm^−1^; NaCl 100–500 mmol L^−1^; treatment time NR	Direct electric-field-induced anisotropic gelation	Gel-phase fraction of 36.80–62.17%; hardness of 96.13–210.54 g; springiness of 33.88–68.30%; water-holding stability decreased from 99.62% to 66.75%	Increasing ionic strength strengthened the gel but compromised water-holding stability, demonstrating a structure–function trade-off	[49]
Low-voltage direct-current electric field	Soy protein isolate/konjac glucomannan (SPI/KGM)	2.98 V cm^−1^; KGM 1.0–2.5%; treatment time NR	Electric-field-directed formation of a double-network hydrogel	With increasing KGM, transverse hardness decreased from 76.62 to 41.43 g and longitudinal hardness from 60.03 to 34.35 g; water-holding stability increased from 69.64% to 90.12%	Formulation and field effects were strongly coupled; denser organization did not correspond to greater hardness	[42]
Ohmic heating/moderate electric field (MEF)	Potato protein isolate (9 wt%, pH 7, and 25 mM NaCl)	3–24 V cm^−1^; 25→65 °C; come-up time of 240 or 1200 s; holding time of 30 or 600 s	Electrothermal gelation	Ohmic heating produced lower protein denaturation, lower water-holding capacity, lower rigidity, and more gap-like/frayed networks than conventional heating under the tested conditions	Higher field exposure did not universally improve gel properties; thermal history and electric-field effects must be separated	[55]
Ohmic heating/moderate electric field (MEF)	Egg white protein (9.8 wt% protein, 0.395 wt% NaCl, and pH 7)	25→85 °C; come-up time of 240 or 1200 s; holding time of 30 or 900 s; field strength varied; specific energy NR	Electrothermal heat-induced gelation	Ohmic heating altered ovalbumin denaturation, intermolecular β-sheet organization, hydrophobic interactions, network morphology, and macroscopic gel properties relative to conventional heating	Differences between ohmic and conventional heating cannot be interpreted as purely nonthermal unless heating history and electrochemical effects are rigorously matched	[56]
Pulsed electric field (PEF)	Alginate–fish gelatin matrix	14 kV cm^−1^; 900 Hz; monopolar square waveform; 58% duty ratio; 6 min; electrode spacing of 2.5 cm	PEF-assisted protein–polysaccharide crosslinking	Particle size of ~80 nm versus 250–550 nm after homogenization; porosity of 0.0973 versus 0.2366 cm^3^ g^−1^; mineral entrapment of 21→33%; vitamin entrapment of 15→24%	PEF produced a more compact structure and improved entrapment; direct comparison with homogenization does not fully isolate electrical effects	[57]
Pulsed electric field (PEF)	Chickpea flour slurry (10%, w/w)	1 or 2 kV cm^−1^; 410–500 kJ kg^−1^; effective treatment time of 44.4–227.4 ms; followed by 24 h at 4 °C	PEF-induced heating/structuring followed by cooling	Hardness of 0.35 ± 0.01→2.94 ± 0.19 kPa; G′ of 195 ± 42→2350 ± 466 Pa; yield stress of 5.7 ± 5.6→423.4 ± 135.2 Pa	No significant rheological/textural difference between 1 and 2 kV cm^−1^; gelation was mainly associated with Joule-driven starch gelatinization, with protein aggregation becoming relevant above ~450 kJ kg^−1^	[58]
Low-frequency magnetic field (LF-MF)	Potato protein–linseed oil emulsion gel	0–10 mT; optimum response reported at 6 mT; exact frequency and treatment time NR in the reported paper	Magnetic-field-assisted emulsion gel formation	At 6 mT, gel strength increased from 0.33 to 0.61 N·mm and WHC from 42.7% to 46.3%; network became denser and more homogeneous	Maximum performance occurred at an intermediate field intensity rather than increasing monotonically across the tested range	[37]
High-pressure processing (HPP)	Pea protein (15%) + κ-carrageenan (0.1–1.0%)	100–600 MPa; 5–30 min	Direct pressure-induced composite gelation	At 1% κ-carrageenan and 600 MPa, compressive strength was ~27-fold that of HPP-treated pea protein alone and >5-fold that of the heat-induced counterpart	The useful pressure window depended strongly on polysaccharide concentration and the pressure–time combination	[41]

Abbreviations: HIU, high-intensity ultrasound; MEF, moderate electric field; PEF, pulsed electric field; LF-MF, low-frequency magnetic field; HPP, high-pressure processing; GDL, glucono-δ-lactone; WHC, water-holding capacity; G′, storage modulus; NR, not reported.

**Figure 5:**
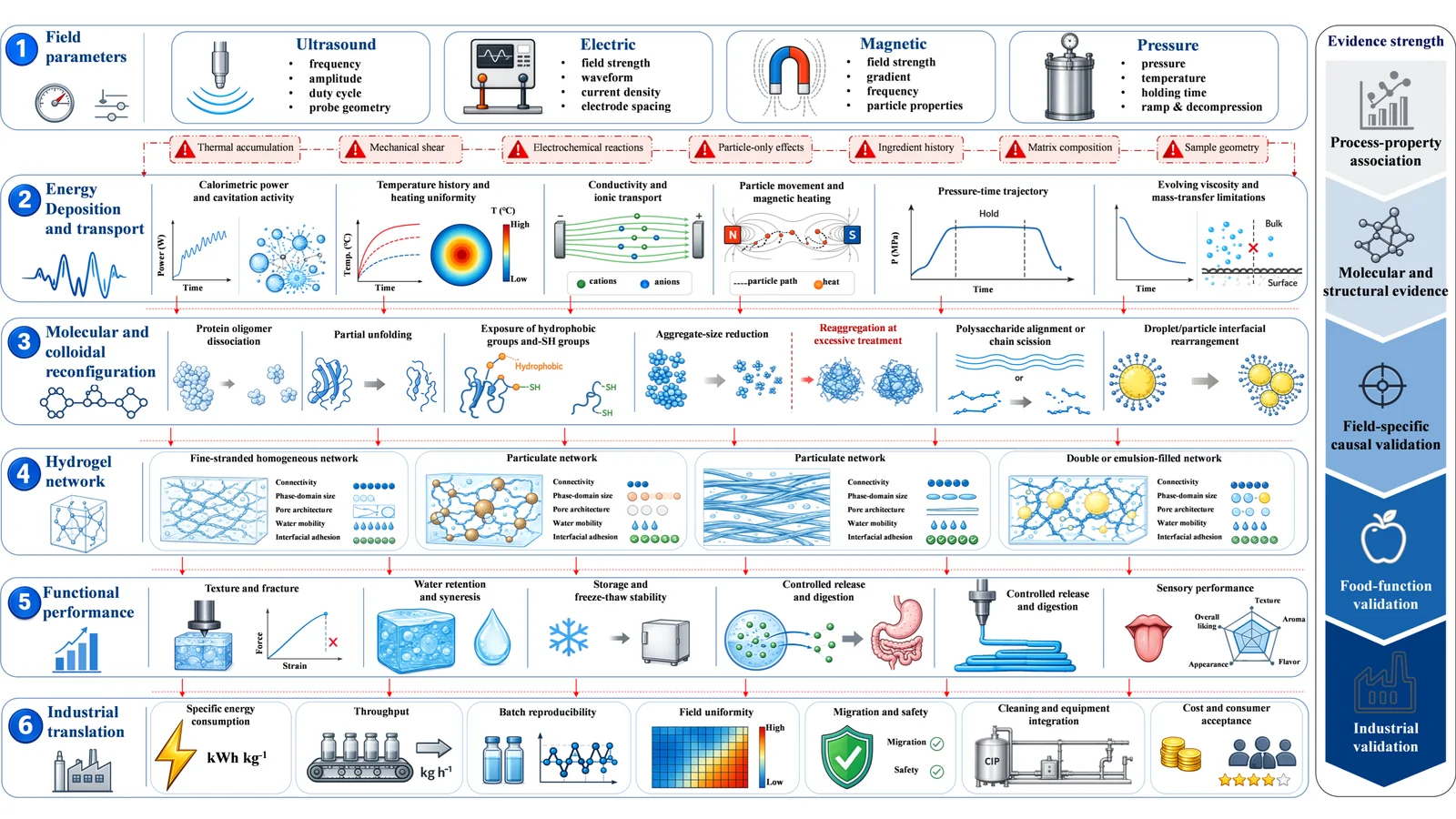
A unified field–process–structure–function framework linking measurable field inputs to hydrogel functionality and translational feasibility.

**Table 2:** Unified comparison of physical-field routes for food hydrogel engineering.

Field Modality/Related Pretreatment	Primary Coupling and Intervention Mode	Key Process Descriptors	Typical Molecular and Network Responses	Major Confounders or Failure Modes	Reference
High-intensity ultrasound	Acoustic cavitation, microstreaming, local shear, transient heating, and radical chemistry; mainly structural priming, but direct field-mediated gelation is emerging.	Frequency; probe or transducer geometry; amplitude; duty cycle; treatment time; vessel volume; immersion depth; gas content; calorimetric power; cavitation activity; and temperature history.	Aggregate disruption or reaggregation; exposure of hydrophobic and sulfhydryl groups; altered protein–polysaccharide co-assembly; and finer particulate, double-network, nanocellulose, or emulsion-filled architectures.	Strong spatial nonuniformity near probes; attenuation at high viscosity and solids; local heating; radical-mediated protein or lipid oxidation; chain scission; and over-aggregation and syneresis.	[27,50,59]
Low-voltage direct-current electric field	Electrophoretic migration, electro-osmosis, polarization, and directional assembly during gel formation.	Field strength; current density; electrode material and spacing; polarity; treatment duration; conductivity; pH profile; temperature; and sample geometry.	Spatial concentration gradients; aligned protein or polysaccharide strands; anisotropic single or double networks; and direction-dependent texture, swelling, and water transport.	Electrode-induced pH gradients; gas evolution; metal-ion release; local gelation; field inhomogeneity; relaxation of alignment after field removal.	[24,49]
Ohmic or moderate-electric-field heating	Volumetric Joule heating combined with polarization and ionic transport; field-mediated thermal gelation.	Voltage gradient; waveform and frequency; current; electrical conductivity; electrode configuration; heating rate; temperature distribution; holding time; and total electrical energy.	Accelerated and potentially more uniform heating; protein-specific changes in unfolding, aggregation, particle size, β-sheet formation, intermolecular interactions, and water retention.	Thermal history is inseparable unless rigorously matched; electrode reactions, gas formation, local pH shifts, fouling, and conductivity drift may dominate the response.	[32,55,56]
Pulsed electric field (PEF)	Short high-voltage pulses cause polarization, electroporation-like effects, aggregate restructuring, and limited thermal exposure; mainly structural priming before later gelation.	Field strength; pulse width; pulse number; pulse frequency; specific energy; inlet/outlet temperature; conductivity; and treatment chamber and flow conditions.	Smaller or reorganized particles; altered charge distribution and protein association; enhanced subsequent ionic, thermal, acid, enzymatic, or pressure crosslinking; and improved entrapment in selected systems.	Temperature and hydrodynamic shear may be confounded with pulse effects; precursor changes may be erased during subsequent gelation; and excessive treatment can promote aggregation.	[57,60]
Magnetic field with responsive inclusions	Magnetic force and torque, particle chain formation, local compaction, and magnetothermal activation; structuring, post-gel actuation, or active food-contact coating.	Field strength and gradient; frequency; exposure time; magnet distance; and magnetic particle identity, size, coating, concentration, distribution, and heating profile.	Particle alignment or chaining; localized densification; remote heating or release; enhanced formation of selected emulsion gels; and responsive antimicrobial coatings.	Native food biopolymers are weakly magnetic; particle-only effects, aggregation, color, grittiness, thermal contributions, migration, and regulatory uncertainty can dominate.	[37,38]
High hydrostatic pressure (HPP)	Isostatic compression shifts volume-dependent equilibria and perturbs noncovalent interactions; structural priming or direct pressure-induced gelation.	Pressure; ramp and decompression rate; holding time; starting and actual temperature; pressure–time integral; protein concentration; pH; and salts and co-solutes.	Oligomer dissociation, hydration changes, partial unfolding, and reassembly into fine-stranded, particulate, phase-separated, or composite networks; water redistribution and densification.	Adiabatic heating; post-decompression aggregation; over-compaction; brittle junctions; water expulsion; and strong dependence on pH, concentration, and co-solutes.	[40,41]
Related mechanical pretreatment: High-pressure homogenization (HPH)	Dynamic shear, turbulence, impact, and cavitation in a narrow valve; structural priming before acid, thermal, or enzymatic gelation.	Valve pressure; number of passes; inlet/outlet temperature; flow rate; valve geometry; specific energy; and particle-size distribution.	Particle-size reduction, altered solubility and interfaces, and denser subsequent gels at an intermediate processing severity.	Mechanistically distinct from HPP; excessive passes or pressure can reaggregate proteins and increase temperature.	[42,43]

## 3. Acoustic-Field-Mediated Reconfiguration of Food Hydrogel Networks

### 3.1. Cavitation Dynamics, Microstreaming, and Local Energy Dissipation

At the hydrogel level, the relevance of acoustic processing lies in how reactor-dependent energy deposition alters precursor dispersion and subsequent network formation. Probe systems concentrate acoustic energy near the sonotrode, whereas baths and plate transducers generally distribute lower intensities over larger volumes. The resulting balance among mechanical disruption, heat generation, and sonochemical reactions is further modified by viscosity, solids content, dissolved-gas content, and temperature [27,61].

Cavitation can fragment large insoluble aggregates into smaller particles, thereby increasing the number and improving the spatial distribution of potential network-forming units. Cavitation may also promote secondary aggregation by increasing collision frequency and exposing previously buried hydrophobic surfaces. Ultrasound-induced gel-like behavior has been demonstrated in almond-based milk with increasing acoustic intensity, accompanied by changes in protein structure and the development of a more organized network [28]. This example is informative because it combines structural modification with microbial inactivation and enzyme-related effects, while also highlighting the difficulty of attributing gelation to a single mechanism. Protein conformational modification, heat accumulation, particle–particle interactions, and changes in the local concentration of dispersed components may all contribute to the observed gelation response.

### 3.2. Protein Conformational Transitions, Aggregation Pathways, and Gel Network Formation

Across protein systems, moderate sonication most consistently modifies the size and reactivity of precursor aggregates before subsequent gelation, but the magnitude and direction of the response are protein-specific. Early studies on soy protein associated ultrasound pretreatment with increased gel strength and water-holding capacity, whereas subsequent work involving soy, pea, potato, oat, faba-bean, and aquatic proteins has shown that comparable nominal treatments do not necessarily generate equivalent network outcomes [62,63].

The initial physicochemical state of the ingredient may exert greater influence on the treatment response than the nominal ultrasound settings alone. Ultrasound-extracted faba-bean protein exhibited distinct in situ gelation and water-holding behavior relative to commercial pea and soy isolates, consistent with compositional and structural differences introduced during extraction and drying [64]. Soy protein gels with different 11S/7S ratios likewise exhibited distinct responses, and treatment conditions that increased firmness could simultaneously reduce water-holding capacity [65]. These findings argue against the generalized claim that ultrasound uniformly improves the properties of protein gels. Ultrasound alters the accessible aggregation pathway, but whether the resulting pathway is desirable depends on protein subunit composition, insoluble particle content, protein concentration, and the mechanism used to trigger subsequent gelation.

Direct acoustic-field-mediated gelation is emerging as an alternative to the conventional use of ultrasound solely as a pretreatment. High-intensity ultrasound has generated gel-like networks in plant-based beverages, and sonication combined with acidification can reduce reliance on conventional thermal treatment [28,29]. Mechanistic attribution, however, remains uncertain in a substantial part of the literature because ultrasound inevitably couples cavitation-driven shear and sonochemical effects with bulk and localized heating. Many studies compare sonicated samples with untreated controls or with conventionally heated samples that do not reproduce the same heating rate, temperature–time trajectory, or spatial–temperature gradients. In such experimental designs, enhanced unfolding, aggregation, or gel strength should be interpreted as treatment-associated rather than unequivocally field-specific. This limitation is illustrated by Yuan et al. [29], who demonstrated that heat accumulation during high-intensity ultrasound itself promoted soy protein unfolding and subsequent GDL-induced network crosslinking. Stronger causal evidence, therefore, requires thermally matched controls, continuous temperature monitoring, and, where feasible, calorimetric power and cavitation measurements to separate thermal contributions from mechanical and sonochemical effects.

### 3.3. Remodeling Protein–Polysaccharide Co-Assembly and Phase Behavior

Protein–polysaccharide systems offer a broader compositional and structural design space than single-protein gels, but they also introduce a greater number of competing assembly and phase separation mechanisms. Ultrasound can improve the dispersion of both components, reduce protein aggregate size, modify polysaccharide conformation, and alter protein–polysaccharide complexation kinetics. Moderate sonication may promote the formation of finer bicontinuous or interpenetrating networks, whereas excessive treatment can depolymerize polysaccharides, intensify protein aggregation, or drive the system toward macroscopic phase separation. The order of component addition is important because a polysaccharide present during sonication can shield interfaces, alter local cavitation behavior, or bind to newly exposed protein interaction sites [28,66].

Recent studies illustrate that the effects of ultrasound are function-dependent rather than uniformly strengthening. Ultrasound treatment combined with curdlan-reinforced pea protein gels is designed for dysphagia management by promoting a more stable network supported by noncovalent interactions [67]. Ultrasound-extracted soy protein combined with arabinoxylan formed a tough, pH-responsive double-network hydrogel in which compositional variation shifted riboflavin release behavior from a more Fickian regime toward nonlinear diffusion [68]. Soft rice protein–starch gels containing ultrasound-modified protein exhibited greater structural homogeneity and reduced starch retrogradation without compromising water retention [69]. Collectively, these examples show that ultrasound can regulate protein–polysaccharide co-assembly while also demonstrating that the desired functional endpoint may prioritize softness, controlled release, or storage stability rather than maximum mechanical strength.

### 3.4. Engineering Nanocellulose, Particle-Stabilized, and Emulsion-Filled Hydrogel Architectures

Nanocellulose fibrils and protein particles provide high-aspect-ratio and interfacially active building blocks for hierarchical hydrogel design. Ultrasound can individualize bundled fibers, modify fibril length distribution, disperse particles, and enhance particle or protein adsorption at oil–water interfaces. Ultrasound-induced nanocellulose hydrogels have been applied as structuring and stabilizing matrices for high-internal-phase Pickering emulsions [70]. In emulsion-filled gels, ultrasound may be applied before emulsification, after droplet formation, or selectively to the protein phase before emulsion assembly. These processing routes generate distinct droplet size distributions, interfacial compositions, and network crosslink densities. Sonication of a preformed emulsion can improve droplet packing and protein-mediated bridging, but it may also induce recoalescence if the protective interfacial layer is disrupted [71].

The central design challenge is to coordinate two coupled structural networks: the continuous hydrogel matrix and the dispersed droplet- or particle-based network. Droplets function as active fillers when they are strongly coupled to the surrounding matrix and deform cooperatively with it; they act as structural defects when interfacial adhesion is weak. Ultrasound can, therefore, modulate bulk mechanical behavior through the combined effects of protein modification within the continuous phase and restructuring of the droplet–matrix interface [72]. Mechanistic studies should report droplet-size distribution, interfacial composition, oil release, nonlinear rheological behavior, and fracture properties rather than relying solely on small-amplitude linear viscoelastic moduli.

### 3.5. Encapsulation, Molecular Transport, and Gastrointestinal Functionality

Field-modified hydrogels are widely investigated as delivery matrices because network pore architecture, water mobility, and available interaction sites govern the retention and release of encapsulated compounds. Ultrasound can improve the initial dispersion of poorly soluble compounds and enhance encapsulation by exposing additional hydrophobic or charged binding regions within the matrix [73]. Conversely, ultrasound can accelerate release by producing a more open network or reducing polymer molecular weight through chain scission. Performance should, therefore, be evaluated not only through encapsulation efficiency but across the linked sequence of processing stability, retention during storage, release in the oral phase, resistance under gastric conditions, intestinal disassembly, and eventual bioaccessibility (Figure 6A) [74,75].

The dominant transport mechanism can shift with changes in network composition and architecture. In the soy protein/arabinoxylan double network, increasing arabinoxylan content altered riboflavin release kinetics, indicating a coupling between network toughening and increased resistance to molecular transport [68]. Ultrasound-assisted emulsion-filled pectin hydrogels have been used to deliver vitamin mixtures, illustrating how a dispersed lipid phase can protect hydrophobic compounds, while the continuous hydrogel matrix regulates transport through the aqueous phase [66]. Digestion studies should monitor network erosion, swelling, enzymatic cleavage, droplet lipolysis, and the chemical transformation of encapsulated compounds. Static release tests in simple buffer systems cannot substitute for validation under physiologically relevant gastrointestinal conditions.

### 3.6. Ultrasound Dose–Structure–Function Trade-Offs and Oxidative Constraints

Because nominal ultrasound settings are not transferable across equipment, practical processing windows should be defined using calorimetric or absorbed energy together with cavitation activity and temperature history. Spatial heterogeneity must also be considered in concentrated pre-gel systems because the region adjacent to the probe can experience substantially different acoustic conditions from the bulk material [65,76,77].

Oxidative constraints require greater consideration when defining practical ultrasound-processing windows. Acoustic cavitation can generate reactive oxygen species, including highly reactive radicals arising from sonochemical reactions, while vigorous mixing and interfacial renewal may increase oxygen incorporation into the matrix. These effects are particularly relevant in lipid-containing and emulsion-filled hydrogels, where sonication can promote both protein oxidation and lipid peroxidation. For example, ultrasound caused greater protein oxidation in a myofibrillar protein–soybean oil system than in the corresponding oil-free protein system, indicating that lipid-containing matrices may be more susceptible to coupled oxidative reactions [78]. Excessive sonication may, therefore, increase protein carbonyl formation, consume sulfhydryl groups, accelerate lipid oxidation, and degrade oxidation-sensitive nutrients even when desirable rheological or structural changes are obtained. Food safety evaluation should consequently include appropriate oxidation markers, such as protein carbonyls, free sulfhydryl groups, primary or secondary lipid oxidation products, and the stability of oxidation-sensitive nutrients, rather than relying solely on gel strength or water-holding capacity. Processing windows should be defined by jointly considering structural functionality, oxidative stability, sensory quality, and the chemical safety of the final food product [66].

Taken together, the ultrasound literature does not support a universal dose–response relationship. Moderate treatment can improve dispersion and network connectivity, whereas higher energy deposition may intensify reaggregation, oxidation, or water loss; the crossover between these regimes shifts with protein source, concentration, subunit composition, and prior processing history. The quantitative conditions summarized in Table 1 should, therefore, be regarded as matrix-specific operating windows rather than directly transferable ultrasound optima.

## 4. Electric-Field-Directed and Magnetically Addressable Hydrogel Systems

### 4.1. Electric-Field-Directed Assembly and Anisotropic Hydrogel Formation

Electric fields offer a route to directional structuring under high-moisture conditions. Charged proteins and polysaccharides may undergo electrophoretic migration, whereas dipolar species and elongated assemblies can orient relative to the applied field. Low-voltage direct-current processing has been used to produce anisotropic soy protein hydrogels at approximately 1.5 V cm^−1^, with salt concentration influencing strand thickness, pore architecture, mechanical properties, swelling behavior, and water retention stability [49]. These studies indicate that field-induced anisotropy is not solely a visually apparent structural feature but can also produce direction-dependent functional behavior. The applied electric field can alter spatial concentration profiles and molecular organization, whereas ionic conditions govern species mobility and the extent of electrostatic screening. The principal mechanistic challenge is to distinguish field-induced alignment from electrode-mediated pH gradients and spatially localized gelation. Rigorous evaluation requires quantitative orientation analysis, longitudinal and transverse mechanical testing, chemical mapping along the electrode axis, and assessment of structural persistence after field removal [79,80]. In food design, anisotropy should be linked to mastication, fibrous perception, directional fluid transport, or controlled disassembly rather than being reported solely as an isolated structural characteristic.

### 4.2. Electrothermal Gelation Under Ohmic Heating: Separating Thermal and Field-Specific Effects

Ohmic heating generates heat volumetrically within a conductive sample and can shorten heating come-up time and improve bulk temperature uniformity. Moderate electric fields applied during ohmic heating have been reported to alter protein aggregation, particle-size distribution, intermolecular interactions, and gel properties in whey, pea, egg white, potato, and soy protein systems [32]. The direction and magnitude of these responses are not consistent across protein systems or processing conditions. Pea protein gels produced under appropriately selected moderate electric fields exhibited greater microstructural uniformity and higher water-holding capacity than conventionally heated controls [32]. Studies of potato protein and egg white reported reduced denaturation or changes in β-sheet organization and hydrophobic interactions, in some cases producing less rigid gels or gels with lower water-holding capacity [55].

These inconsistent responses are scientifically informative because they indicate that exposure to an electric field does not produce a universal strengthening effect in protein gels. For ohmic heating, mechanistic separation is particularly challenging because Joule heating is intrinsic to the treatment. A valid comparison with conventional heating must, therefore, match not only the endpoint temperature but also the heating rate, come-up time, holding period, and overall temperature–time history. Several studies have compared ohmic and conventional heating under controlled thermal protocols [55], yet complete equivalence of the local thermal environment cannot be assumed because conductivity gradients, current density distributions, and electrode-adjacent phenomena may generate spatially heterogeneous thermal and electrochemical conditions. Consequently, differences in protein denaturation, β-sheet organization, hydrophobic interactions, water-holding capacity, or network morphology cannot be assigned solely to a nonthermal electric-field effect unless thermal and electrochemical contributions are independently constrained. The available evidence is, therefore, more appropriately interpreted as demonstrating a matrix- and protocol-dependent electrothermal response, while unequivocal field-specific molecular effects require more rigorous causal validation [18,56,81].

### 4.3. Pulsed Electric Fields as Structural Priming Before Gelation

Pulsed electric fields are most commonly associated with cellular electroporation, but they can also modify dispersed protein systems and protein–polysaccharide assemblies. Short-duration, high-voltage pulses can alter charge distribution, expose functional groups, dissociate aggregates, or promote new intermolecular associations. Bulk heating may remain limited at low specific energy, but thermal contributions can become substantial in conductive matrices or under high-energy treatment conditions. Their most defensible role in hydrogel fabrication is as a structural-priming treatment in which the field modifies a precursor that is subsequently gelled by thermal treatment, ionic crosslinking, acidification, pressure, or enzymatic action [82].

A study applied pulsed electric fields to promote crosslinking in alginate–fish gelatin matrices. Relative to homogenization, the pulsed-electric-field-treated system contained smaller particles and exhibited a more compact, less porous structure; mineral and vitamin entrapment increased from 21% to 33% and from 15% to 24%, respectively [57,83]. PEF has also been applied to texture-modified chickpea flour gels, where the final response was dominated by Joule-driven starch gelatinization within the tested specific energy range [60]. These studies support the structural-priming concept, but establishing direct causal links to the final hydrogel network requires temperature- and shear-matched controls, together with evidence that field-induced precursor modifications persist through the subsequent gelation step.

### 4.4. Conductive Hydrogel Systems for Food Contact Sensing and Emerging Electrical Actuation

Evidence for conductive hydrogel systems is currently strongest in food contact sensing and labeling rather than in directly ingestible field-responsive foods. In this context, conductive food-contact hydrogels can incorporate mobile ions or other conductive components to support electrical sensing, deformation, or signal transduction, whereas direct edible applications require substantially stronger evidence of ingredient suitability and exposure safety. A double-network hydrogel composed of starch, alginate, polyvinyl alcohol, bentonite, and salt has been developed as an environmentally compatible electronic food label with tunable mechanical properties and electrical conductivity [84]. Such systems can conform to irregular surfaces and convert changes in strain, humidity, temperature, or ionic conditions into measurable signals.

Electrically triggered release and deformation remain conceptually attractive for on-demand flavor release, nutrient delivery, and adaptive texture, but these functions introduce substantial material, electrochemical, and regulatory constraints. Electrical current can induce electrolysis, Joule heating, ion migration, and electrode degradation. A conductive filler that is acceptable for use in a food contact sensor may not be suitable for direct incorporation into an ingestible formulation. Food contact applications require migration testing and stable containment of conductive components, whereas ingestible applications require regulatory authorization of all constituents and assessment of exposure dose. Performance should be evaluated after repeated humidity cycling, refrigeration, freezing, mechanical deformation, and contact with acidic or high-salt food matrices [24].

### 4.5. Magnetically Structured and Remotely Responsive Hydrogel Systems for Food Applications

Direct food hydrogel evidence for magnetic-field-mediated structuring remains limited but is beginning to emerge. Low-frequency magnetic field treatment of potato protein emulsion gels and protein-rich food matrices provides direct food-related evidence that magnetic treatment can modify gelation and network organization [37,85]. In contrast, Fe_3_O_4_-containing chitosan-based hydrogel coatings developed for beef preservation should be classified as food contact applications rather than direct edible-hydrogel structuring [38]. Biomedical magnetic hydrogels discussed elsewhere in this review are used only as mechanistic inspiration for particle alignment, remote actuation, or magnetothermal activation. These three evidence contexts should not be considered equivalent because their formulation requirements, exposure routes, safety criteria, and intended functions differ substantially.

The most realistic near-term opportunities may lie in magnetically addressable processing aids, packaging coatings, and sensors rather than bulk ingestible hydrogels [86,87]. Remote magnetic positioning can concentrate active agents at selected interfaces or facilitate the recovery of magnetically responsive processing materials. Magnetothermal heating can provide localized activation while limiting bulk heating of the surrounding product. The realization of these advantages depends on uniform particle dispersion, field distribution, and effective field penetration into the product. Aggregation of magnetic inclusions can cause undesirable darkening, a gritty mouthfeel, localized stiffening, or increased risk of particle migration [44]. Field-responsive systems must, therefore, integrate food quality and safety considerations from the earliest stages of material and process design.

### 4.6. Magnetic Hydrogel Systems Require Stronger Mechanistic Evidence and Safety Validation

For magnetic hydrogel systems, safety and causal validation remain co-limiting constraints. Magnetic responsiveness does not establish food-grade status, and nanoscale Fe_3_O_4_ or related inclusions must be evaluated in the specific particle form and intended exposure route. Relevant assessment should include particle identity and purity, size distribution, surface chemistry, aggregation and dissolution behavior, migration, gastrointestinal transformation, exposure dose, and appropriate toxicological endpoints. Field strength, gradient, frequency, source distance, exposure duration, and temperature history should also be reported because they influence particle organization, migration, and magnetothermal responses [54,88].

Mechanistic and translational claims should be evaluated according to a clearly defined hierarchy of evidence. An increase in gel strength observed in the simultaneous presence of magnetic particles and an applied field establishes a combined treatment association but does not identify the underlying mechanism. Demonstrating magnetic alignment requires quantitative orientation analysis or spatial mapping of particle organization. Establishing a field-specific molecular effect requires particle-only, field-only, full-treatment, and matched-temperature controls. Demonstrating food applicability requires sensory evaluation, storage testing, and, depending on the intended exposure route, digestion or migration assessment and repeated-use validation [13,89].

Across electric and magnetic approaches, response magnitude should also be interpreted together with the maturity of causal evidence. Low-voltage direct-current processing provides direct evidence for directional and anisotropic network formation, whereas ohmic-heating responses are more appropriately interpreted as coupled electrothermal effects unless thermal and electrochemical contributions are rigorously controlled. PEF is currently better supported as a precursor-structuring treatment than as an independent gelation route. Magnetic-field approaches remain supported by fewer direct edible-food studies. Quantitative comparison, therefore, requires consideration of both the magnitude of the reported property change and the strength of evidence linking that change to the applied field.

## 5. Pressure-Mediated Network Reconfiguration and Hybrid-Field Processing

### 5.1. Pressure-Induced Molecular Transitions and Interaction Rearrangement

Unlike acoustic and electric-field treatments, high-pressure processing acts through nearly uniform isostatic compression rather than strongly localized energy deposition. Pressure shifts volume-dependent equilibria, promoting oligomer dissociation, hydration changes, partial unfolding, and pressure-history-dependent reassembly while leaving most covalent bonds comparatively resistant [40,90]. Polysaccharide responses are generally less dominated by covalent structural change and instead involve alterations in hydration, crystallinity, chain association, and protein–polysaccharide interactions.

Pressure-induced gelation, therefore, encompasses multiple molecular and colloidal pathways rather than a single universal mechanism. Fine-stranded networks are favored when electrostatic or steric repulsion maintains partially unfolded molecules in a dispersed state, allowing them to connect through relatively uniform intermolecular junctions. Particulate networks are more likely to form when attractive interactions and proximity to the protein isoelectric point promote extensive aggregation before or during network percolation. Composite systems introduce additional contributions from excluded-volume interactions, phase separation, and active or inactive filler behavior. Spectroscopy can identify conformational changes, but dissolution tests, selective interaction–perturbation experiments, microscopy, and rheological analysis are required to determine which interactions stabilize the final network architecture [91,92,93].

### 5.2. Pressure-Controlled Gelation in Plant Protein and Composite Systems

Dairy and muscle proteins provided the earliest demonstrations of pressure-induced gelation, whereas plant protein systems are now receiving increasing research attention. Pea protein can form self-supporting gels directly during high-pressure treatment (Figure 6B) [94]. In one systematic study, pH shifting before high-pressure treatment was also reported to improve the freeze–thaw stability of the resulting gels. These findings indicate that high-pressure treatment can generate product-relevant textures ranging from pudding-like to tofu-like structures without reliance on conventional heat-induced gelation. The incorporation of κ-carrageenan substantially broadened the pressure gelation processing window [95]. Pea protein gels containing 1% κ-carrageenan and treated at 600 MPa exhibited a 27-fold increase in compressive strength relative to pressure-treated pea protein alone and more than a fivefold increase relative to heat-induced counterparts [96]. The polysaccharide formed finely dispersed domains within a compact, pressure-modified protein matrix, thereby compensating for the relatively limited capacity of pea protein to form disulfide crosslinks. This example illustrates that pressure conditions and formulation must be co-designed: pressure establishes the molecular and colloidal state, whereas the secondary network or filler phase translates that state into macroscopic mechanical performance (Figure 6C) [97].

**Figure 6:**
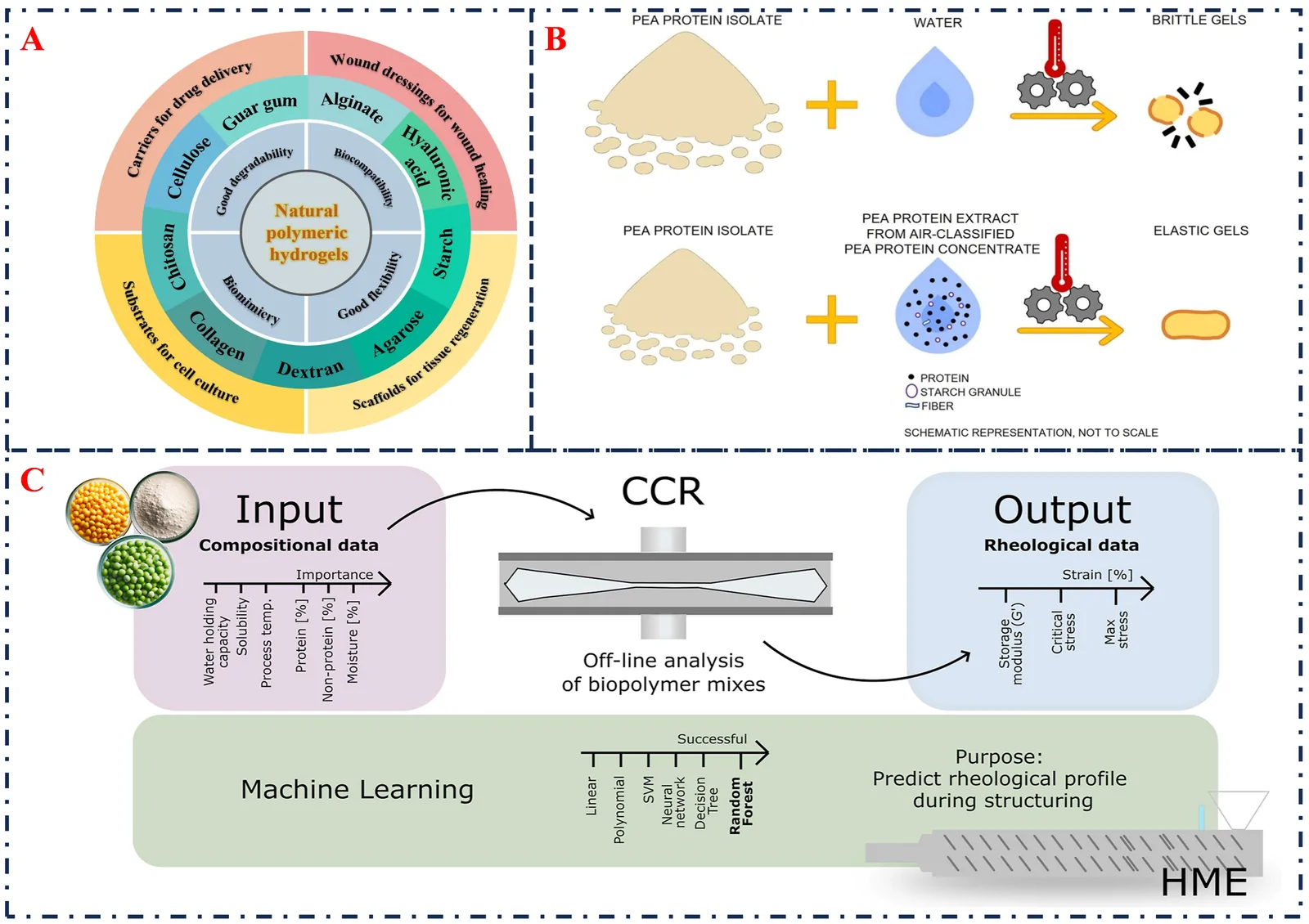
Representative examples illustrating different stages of functional translation and hydrogel design. (**A**) The application landscape of natural-polymer hydrogels provides broader mechanistic and translational context but is not treated as direct evidence for edible food hydrogel structuring [98]. (**B**) Pea protein gelation illustrates how native protein composition and ingredient state influence the capacity to form elastic food networks [94]. (**C**) A machine learning workflow for plant protein–polysaccharide mixtures illustrates how formulation data can be mapped to rheological outcomes for predictive hydrogel design [96].

### 5.3. Pressure-Driven Water Redistribution, Phase Reorganization, and Network Densification

Water redistribution is central to the structural and functional performance of pressure-induced gels. Pressure modifies molecular hydration and intermolecular distances during treatment, whereas decompression may immobilize water within newly formed pores or promote its expulsion from over-compacted domains. Protein–polysaccharide phase separation may become finer when pressure-induced unfolding and aggregate size reduction improve dispersion, but it may become coarser if attractive interactions intensify following decompression. Low-field nuclear magnetic resonance, magnetic resonance imaging, differential scanning calorimetry, and centrifugation-based water-holding assays provide complementary information on water state, spatial distribution, thermal behavior, and bulk retention. Total water-holding capacity alone cannot distinguish among tightly bound, immobilized, and freely mobile water populations (Figure 7) [41,99].

Network densification improves mechanical strength only when it increases connectivity without generating brittle junctions, excessive heterogeneity, or poorly accessible water domains. In pressure-treated pea protein/κ-carrageenan gels, uniformly distributed submicron polysaccharide domains within a compact protein network were associated with enhanced compressive strength [96]. In other systems, excessive pressure intensity or solids concentration may instead promote heterogeneous aggregation and localized structural defects. Mechanical characterization should, therefore, include fracture strain, toughness, creep behavior, and cyclic recovery in addition to hardness and small-amplitude storage modulus.

**Figure 7:**
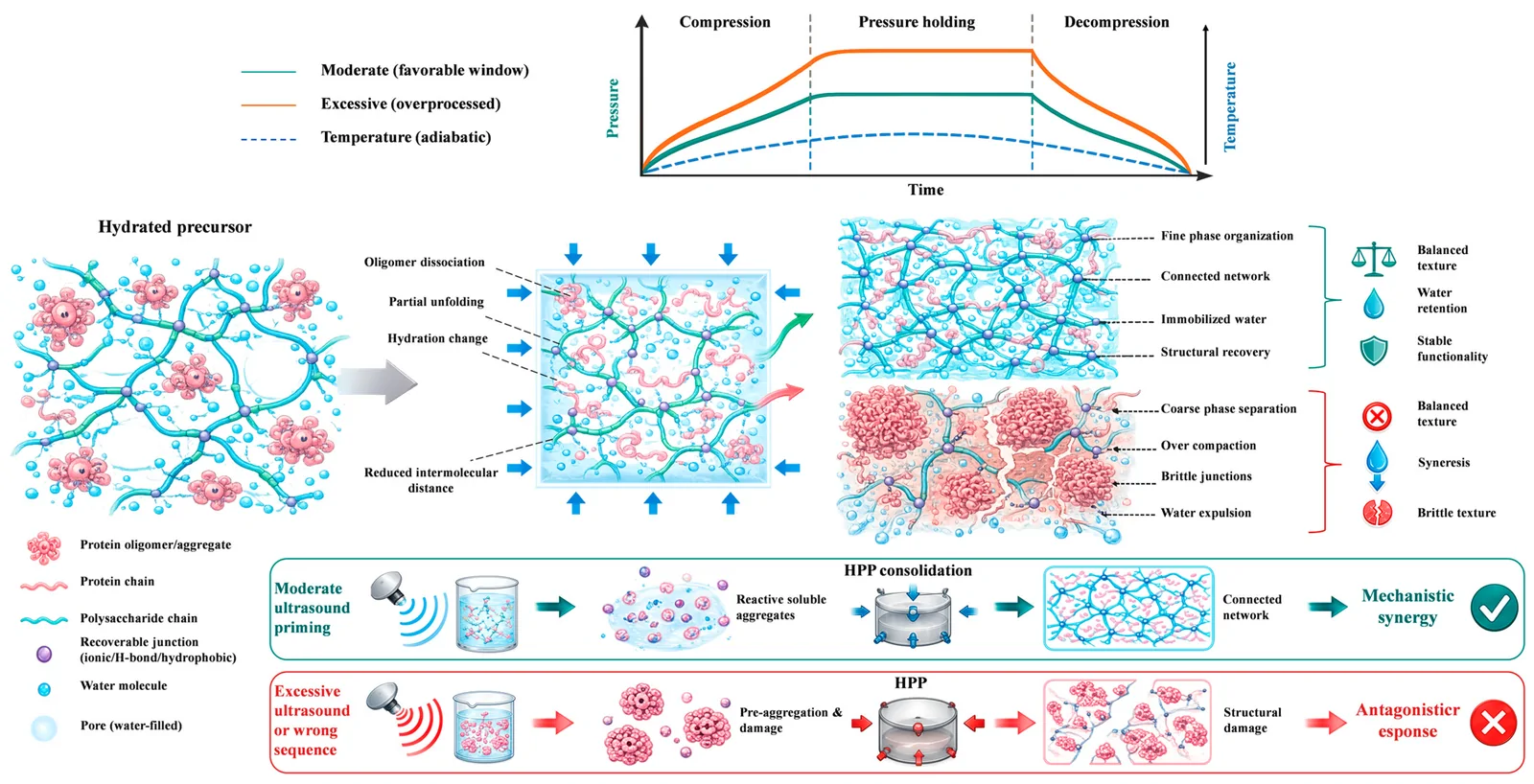
Pressure-history- and treatment-sequence-dependent regulation of water redistribution and network fate in food hydrogels.

### 5.4. Pressure–Temperature–Time Interactions and Processing Windows

Pressure level, treatment temperature, and holding time jointly define an interacting processing window. Moderate pressure may dissociate oligomeric assemblies or structurally prime proteins for subsequent gelation. Higher pressure levels and longer holding periods can promote more extensive unfolding and direct pressure-induced network formation. Adiabatic heating during compression and the initial product temperature jointly determine the actual pressure–temperature trajectory experienced by the sample. A nominally nonthermal treatment may, therefore, reach temperature ranges in which thermal and pressure effects act cooperatively. Reporting only the maximum pressure omits critical descriptors, including compression rate, decompression rate, actual temperature history, and the integrated pressure–time exposure [100].

The optimum pressure–temperature–time combination depends on the targeted product and process outcomes. A brief high-pressure treatment may better preserve fresh flavor while improving dispersion, whereas a longer holding period may be required to achieve direct gelation or the intended level of microbial inactivation. Excessive pressure intensity or holding time can produce over-compacted networks with poor structural recovery while increasing equipment utilization, energy demand, and overall processing burden. The processing window should, therefore, be defined by multiple constraints, including the required microbial lethality where applicable, texture, water retention, sensory quality, nutrient stability, and throughput. High-pressure treatment should not be optimized solely to maximize the elastic modulus or any other single mechanical endpoint [101].

Pressure-based studies similarly demonstrate that pressure level alone is insufficient to predict hydrogel performance. Protein concentration, pH, treatment temperature, holding time, and co-gelling components jointly determine whether pressure promotes useful network reinforcement or excessive densification and aggregation. The strong reinforcement observed in pea protein/κ-carrageenan systems, therefore, represents a coupled formulation–pressure processing window rather than a general effect attributable to 600 MPa treatment alone [40].

### 5.5. Sequential and Synergistic Multi-Field Treatments

Hybrid-field processing can combine complementary physical and molecular mechanisms within a sequential treatment strategy. Ultrasound can improve protein dispersion and induce partial unfolding before high-pressure treatment consolidates the modified species into a connected network. Electric fields can orient or redistribute components before thermal or ionic crosslinking immobilizes the field-induced organization. Pressure treatment can alter protein susceptibility to subsequent enzymatic action or pH-induced association. Field-assisted freezing may generate directionally organized pores or cryogel-like architectures. Treatment order is critical because the first intervention alters the molecular, colloidal, and rheological state of the material encountered by the subsequent process [102,103].

Claims of synergy require evidence beyond the observation that a combined treatment outperforms either individual treatment. The expected additive response should be defined in advance, preferably through factorial experiments that control for total energy input or overall processing severity. A synergistic interaction is supported when the combined response exceeds that predicted from the independent effects, whereas antagonism is indicated when one treatment diminishes or reverses the benefit of the other. Mechanistic synergy should also be established; for example, ultrasound may generate soluble, reactive aggregates that subsequent pressure treatment converts into a connected network at a lower pressure than would otherwise be required [104,105].

## 6. From Field-Induced Network Changes to Food Functionality and Scalable Processing

### 6.1. Multiscale Evidence Chain from Molecular Transitions to Sensory Performance

Functional translation should be evaluated using measurements matched to the claimed food application rather than inferred from isolated molecular or structural changes. Hydrated-state imaging and mechanical testing are needed when network organization is central to the proposed function, whereas claims related to fibrous perception, storage stability, controlled release, or digestibility require direct sensory, storage, or gastrointestinal validation (Figure 8A,B) [3,104]. Spectroscopic shifts, reduced particle size, or visually denser microstructures alone should not, therefore, be interpreted as evidence of improved food performance.

### 6.2. Energy-Normalized Benchmarking of Field Efficiency and Network Outcomes

Cross-technology efficiency cannot be inferred from nominal field settings because acoustic amplitude, electric-field strength, and pressure–time conditions represent physically different modes of energy delivery. Benchmarking should, therefore, relate specific energy consumption per unit product to treatment time, throughput, and a predefined functional gain. Modality-specific descriptors remain necessary for interpreting how energy is deposited, but they should not be ranked as equivalent measures of treatment intensity [106].

Published food-processing benchmarks illustrate why process sustainability cannot be inferred from treatment duration or nominal field intensity alone. At a production capacity of 120 L h^−1^ and an equivalent level of microbial inactivation, thermal pasteurization of tomato and watermelon juices required 4.36–4.56 kWh per 120 L, whereas pulsed electric field (PEF) and high-pressure processing (HPP) required 14.30–14.42 and 21.72 kWh, respectively, corresponding to approximately 131–137, 429–433, and 652 kJ kg^−1^ for juices of near-unit density [107]. In contrast, an industrially oriented study of chopped tomatoes reported an energy requirement of 54 kWh t^−1^ (194 kJ kg^−1^) for ohmic heating compared with 156 kWh t^−1^ (562 kJ kg^−1^) for conventional appertization, with overall energy efficiencies of 77% and 25%, respectively [107,108]. These values are not hydrogel-specific and should not be interpreted as universal technology constants because treatment objective, equipment scale, product properties, heat recovery, and auxiliary loads differ among studies. They nevertheless demonstrate that field-assisted processing can be either more or less energy-intensive than conventional heating. Hydrogel studies should, therefore, report specific energy together with throughput and functional gain, such as water retained, gelation time reduced, or a target mechanical response achieved per unit energy, as well as measurement uncertainty and batch-to-batch variability [109,110].

### 6.3. Functional Translation: Texture, Water Management, Stability, Delivery, and Printability

Texture is the most frequently reported endpoint, but physical-field engineering can target a substantially broader range of product functions. Directional electric-field assembly may generate soft, anisotropic networks suitable for plant-based meat analogs or foods designed for individuals with dysphagia. High-pressure processing can tune pea protein gels toward jelly-like, pudding-like, or tofu-like mechanical and sensory properties. Ultrasound may soften a composite while reducing starch retrogradation or, under different formulation and processing conditions, strengthen a network to support safer swallowing. The target response should, therefore, be defined by intended product requirements rather than by a generalized preference for maximum hardness [111,112].

Water management provides a critical link between texture, storage stability, mass transport, and sensory perception. A network with high bulk water-holding capacity may still contain mobile water populations that redistribute during storage. Physical fields can modify pore connectivity, molecular hydration, and phase distribution, thereby affecting syneresis, freeze–thaw stability, juiciness, and the release of water, flavors, or encapsulated compounds. Low-field nuclear magnetic resonance and spatial-imaging methods should be integrated with storage studies to connect water-state measurements with actual redistribution and product stability. Emulsion-filled and particulate hydrogels further expand the design space by introducing lipid retention, interfacial stability, and oxidative protection as additional performance targets [113].

Printability requires shear-thinning behavior during extrusion, rapid structural recovery after deposition, sufficient yield stress to support the printed geometry, and stable adhesion between deposited layers. Machine learning studies of pectin-based inks found that viscoelastic parameters accounted for more than 90% of the model-derived feature importance in predicting filament spreading, with tree-based models achieving strong predictive performance on independent test data [114]. Gaussian process models have also been used to predict food formulation printability and reduce reliance on empirical trial-and-error optimization [115]. Physical-field treatments can modify these rheological descriptors, but printability should be evaluated using geometry-specific printing metrics rather than inferred from apparent viscosity alone.

**Figure 8:**
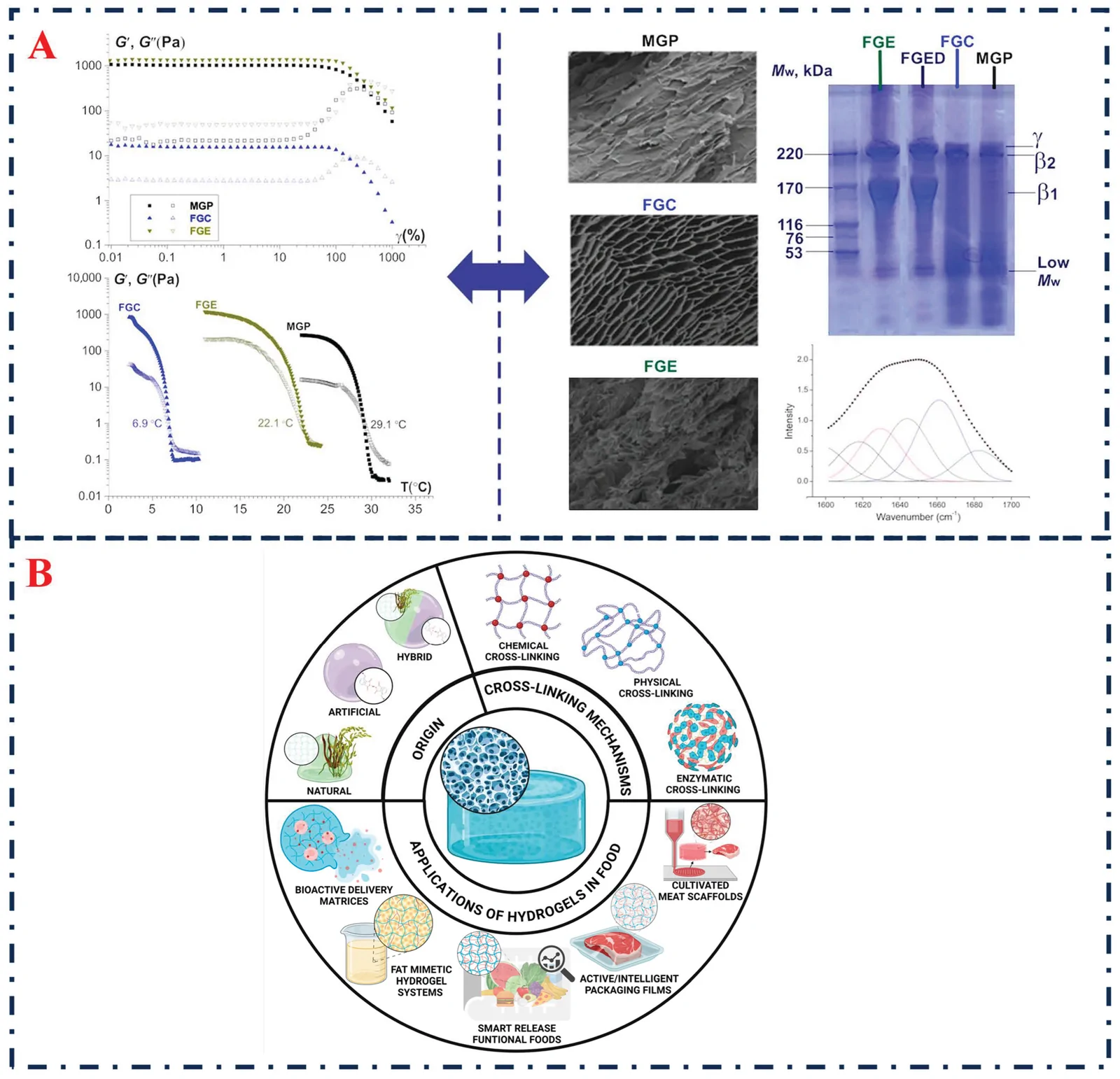
Representative examples illustrating how multiscale characterization and network design support functional translation of food hydrogels. (**A**) Rheological, microstructural, and molecular characterization of fish and mammalian gelatin illustrates the need to connect network mechanics with composition and structure when evaluating practical formulations (The lines in different colors correspond to nicotinamide bands) [116]. (**B**) The broader landscape of food hydrogel crosslinking and applications illustrates how structural design is translated into encapsulation, food packaging, cultivated-food scaffolds, and controlled-release functions [16].

### 6.4. Data-Driven Process–Structure Mapping and Inverse Formulation Design

The number of interacting variables in field-engineered hydrogel systems exceeds what can be efficiently explored through one-factor-at-a-time experimentation. Composition, pH, salt concentration, solids content, temperature, field strength, frequency, pulse mode, treatment duration, reactor geometry, and treatment order may interact nonlinearly. Machine learning models can map these process and formulation inputs onto rheological, structural, and functional response variables. Random forest models predicted linear viscoelastic parameters of plant protein biopolymer mixtures from compositional information, although large-amplitude deformation behavior remained more difficult to predict accurately [117,118]. This limitation is instructive because data-driven models learn statistical relationships represented in the training data and do not automatically recover the physical mechanisms governing fracture, yielding, or structural reorganization.

A robust digital workflow begins with standardized metadata, harmonized measurement protocols, and physically interpretable process descriptors. Nominal field settings should be converted into physically meaningful descriptors such as absorbed energy, temperature history, sample conductivity, cavitation indices, pressure severity, and spatial field gradients. Structural outputs should include particle-size distributions, quantitative orientation metrics, water mobility, network images, and frequency- and strain-dependent rheological spectra. Explainable models can identify which variables contribute most strongly to a prediction, whereas uncertainty quantification can indicate when a formulation lies outside the model’s applicability domain. Inverse design can then search for formulations and processing conditions that satisfy multiple objectives, such as low energy demand, high water retention, specified fracture behavior, and acceptable printability. Recent perspectives on machine learning for food colloids emphasize the need for larger, interoperable datasets, standardized metadata, and physics-informed model constraints [119,120].

### 6.5. Scale-Up and Equipment Integration: Reactor Geometry, Field Uniformity, and Continuous Operation

Most available evidence has been generated in bench-scale batch systems, where short propagation distances and small treatment volumes can mask spatial heterogeneity in field intensity and energy deposition. Scale-up, therefore, cannot be achieved by simply increasing nominal power in proportion to sample volume. In acoustic reactors, increasing propagation distance, solids content, and viscosity attenuates acoustic energy, while standing waves, shielding, and transducer geometry can generate high-intensity and poorly treated regions within the same vessel [59,121]. Part of the delivered acoustic energy is also dissipated as heat rather than cavitation-driven mechanical work. Electric-field systems face a different form of nonuniformity because conductivity gradients, electrode geometry, and evolving composition redistribute current density and Joule heating. Magnetic-field gradients decrease with distance from the field source, whereas high-pressure processing provides comparatively uniform hydrostatic pressure but remains constrained by compression–decompression cycles, batch loading, and auxiliary energy requirements [24,122].

Continuous processing, therefore, requires reactor designs that maintain both field uniformity and a controlled residence-time distribution as the material evolves from a low-viscosity precursor toward a structured or yield stress network. For ultrasound, multi-transducer arrays, frequency modulation, and flow-through reactors can broaden the effectively treated volume, but their performance should be verified by spatial field mapping and calorimetric measurements rather than nominal transducer power alone. Continuous ohmic or pulsed-electric-field systems require controlled electrode spacing, current density distribution, conductivity monitoring, and management of electrode fouling, gas evolution, and local heating. Magnetic systems require reproducible dispersion of responsive particles together with quantitative characterization of the field gradient. High-pressure processing is already industrially established for food preservation, but direct hydrogel manufacture remains predominantly batch-based; semi-continuous configurations are more readily applicable to pumpable precursors than to fully developed gels [27,123].

Industrial evaluation should also distinguish energy delivered by the equipment from energy effectively absorbed by the product. Losses associated with transducers, electrodes, pumping, cooling, compression, and pressure release can become increasingly important at scale. Inline measurements of temperature, conductivity, viscosity, acoustic activity, pressure, and flow rate can support closed-loop control as the material properties change during gelation. Reactor cleanability, avoidance of hygienic dead zones, electrode durability, and compatibility with clean-in-place operation should be treated as design criteria rather than downstream engineering considerations. Accordingly, pilot-scale validation should report field distribution, absorbed or specific energy, residence-time distribution, throughput, and batch-to-batch variability together with the resulting hydrogel properties.

### 6.6. Safety, Regulatory Compliance, Sustainability, and Consumer Acceptance

Physical-field processing does not eliminate chemical, toxicological, or material safety concerns. Ultrasound can promote oxidative modification; electric-field systems may release electrode-derived ions or generate electrochemical reaction products; conductive and magnetic fillers may migrate; and high-pressure treatment may alter allergenicity, digestibility, or the bioavailability of active compounds. Safety evaluation should be conducted using the intended product formulation, processing conditions, and exposure route. Food-contact sensors, recoverable processing aids, and directly ingested hydrogels are subject to different exposure scenarios and regulatory requirements. Material composition, particle form and size, residual reagents, degradation products, migration behavior during storage, and transformation under digestion conditions should be documented [38,84]. For nanoscale magnetic inclusions, regulatory acceptability should be established for the specific particle form and intended exposure route rather than inferred from the status of the corresponding bulk material. For ultrasound-treated foods, safety evaluation should focus on measurable oxidation products and processing-induced compositional changes in the final product, particularly in lipid-rich systems, alongside conventional microbiological and chemical safety criteria.

Accordingly, “green” or “sustainable” should not be treated as intrinsic descriptors of physical-field processing. Comparative life-cycle assessments in food processing demonstrate that environmental performance is strongly dependent on the technology, product, system boundary, energy recovery strategy, and electricity mix. For example, comparative assessment of ohmic and indirect heating reported higher environmental impacts for ohmic heating under the electricity mixes considered for Italy and Australia, whereas substantial reductions in global warming potential were predicted when low-carbon electricity or photovoltaic power was used [124]. Pilot-scale comparisons of thermal, PEF, and HPP pasteurization similarly showed that optimized conventional heating could require less direct processing energy than the two field-based alternatives under equivalent microbial lethality [124]. Conversely, other product- and system-specific assessments have reported energy or environmental advantages for ohmic heating and HPP [125]. At present, however, harmonized LCA data specific to food hydrogel manufacture are insufficient to rank acoustic, electric, magnetic, pressure-based, and conventional gelation routes. Future comparisons should, therefore, define the functional unit and system boundary explicitly and quantify process and auxiliary energy, energy recovery, throughput, water use, cleaning requirements, ingredient inputs, packaging where relevant, and product losses before sustainability advantages are claimed [126,127].

Consumer acceptance depends on the characteristics of the final product and how the underlying technology is communicated. Ultrasound and high-pressure processing already have recognizable applications in the food sector, whereas conductive or magnetic hydrogels may raise concerns regarding particulate additives, technological complexity, or perceived artificiality. Transparent communication, clean-label formulation where technically feasible, and demonstration of acceptable sensory quality are, therefore, important for consumer adoption.

## 7. Conclusions and Outlook

### 7.1. Conclusions

The evidence reviewed here indicates that physical-field engineering has reached markedly different levels of maturity across food hydrogel systems. Ultrasound currently has the broadest experimental basis for modifying protein aggregation, protein–polysaccharide co-assembly, particulate networks, and emulsion-filled gels. Its ability to restructure hydrogel precursors is well demonstrated, although the extent to which these changes arise from cavitation-specific effects rather than heat accumulation and sonochemical reactions remains insufficiently resolved in many studies. High-pressure processing also has comparatively strong support for direct gelation and composite network reinforcement, particularly in concentrated protein and protein–polysaccharide systems, but its outcomes remain highly dependent on pressure–temperature–time history and formulation.

Electric-field approaches occupy an intermediate level of maturity. Low-voltage fields have demonstrated directional and anisotropic network formation, while ohmic heating can modify protein gelation through coupled electrical and thermal effects. However, field-specific molecular contributions remain difficult to establish independently of Joule heating, ionic transport, and electrode reactions. Pulsed electric fields are currently better supported as structural-priming treatments than as independent gelation routes. In contrast, magnetically responsive and electrically conductive edible hydrogels remain at an earlier stage of development. Much of the mechanistic evidence for these systems is still derived from biomedical, sensing, or food-contact materials, and direct validation in ingestible food matrices remains limited.

Across all modalities, the available literature does not support a universal relationship between treatment intensity and improved hydrogel functionality. Beneficial effects occur within matrix- and application-specific processing windows in which molecular activation, transport, and network connectivity are balanced against aggregation, oxidation, phase separation, densification, or water loss. The practical value of physical-field processing, therefore, lies not in maximizing structural modification, but in controlling network assembly reproducibly under conditions that deliver a defined food function with acceptable energy demand, safety, and process complexity.

### 7.2. Outlook

Future progress in physical-field engineering of food hydrogels will depend on converting the currently fragmented body of evidence into transferable and mechanistically defensible design rules. A major challenge is to distinguish genuine field-specific effects from thermal, mechanical, electrochemical, and particle-mediated contributions. This requires matched controls, time-resolved measurements, and direct characterization of energy deposition, particularly for ultrasound and ohmic processing, where heat generation is intrinsically coupled to treatment. Greater consistency in reporting is also needed. Physically meaningful descriptors such as absorbed or specific energy, temperature history, electrical conductivity, cavitation activity, pressure trajectory, spatial field distribution, and relevant matrix properties should be linked to both beneficial and deteriorative responses so that processing windows can be compared across studies more reliably.

The next stage of development should also move beyond simplified protein and polysaccharide model systems toward compositionally realistic foods containing salts, sugars, lipids, insoluble particles, and other components that modify both field coupling and network formation. This transition is particularly important for magnetic and conductive systems, where direct evidence in edible formulations remains limited and food-grade status, migration, gastrointestinal fate, exposure, and toxicological safety require stronger validation. At the same time, promising laboratory-scale responses must be tested under pilot-scale conditions that reflect spatial field heterogeneity, residence-time distribution, evolving rheology, throughput, cleanability, and batch-to-batch reproducibility.

Ultimately, technological value will depend on whether improved hydrogel structure can be translated into reproducible food functionality under realistic processing constraints. Energy efficiency, life-cycle performance, sensory quality, safety, and equipment integration should, therefore, be evaluated together with rheological and structural outcomes rather than treated as downstream considerations. Future high-performance systems are likely to rely on carefully sequenced, moderate-intensity interventions that prepare, orient, crosslink, or stabilize hydrogel networks while limiting thermal, oxidative, mechanical, or electrochemical damage. The objective should not be to maximize structural modification, but to achieve controllable and application-specific functionality with demonstrable process feasibility.

## Figures and Tables

**Figure 2:**
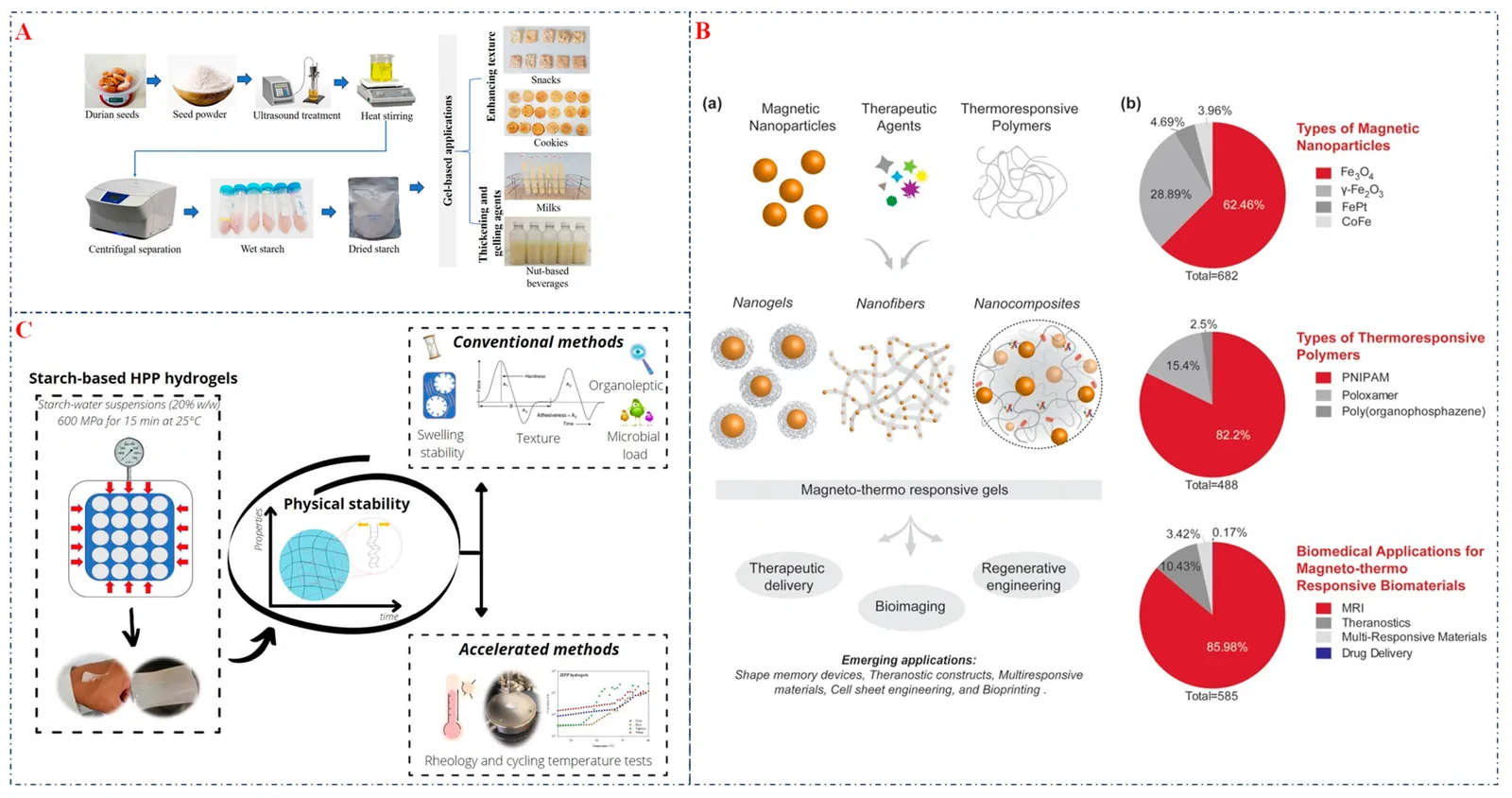
Representative examples of physical-field interactions with hydrogel systems. (**A**) Ultrasound-modified durian starch illustrates direct food-related acoustic modification of a gel-forming biopolymer [20]. (**B**) Magnetic-responsive nanoengineered hydrogels are included as mechanistic inspiration from a biomedical system to illustrate magnetic-particle-mediated field coupling rather than as direct evidence for edible food hydrogels [21]. (**C**) High-pressure-processed starch hydrogels illustrate direct food hydrogel evidence for pressure-mediated network stabilization [22].

## Data Availability

The authors declare that the data supporting the findings of this study are available within this paper. Should any raw data files be needed in another format, they are available from the corresponding author upon reasonable request. No datasets were generated or analyzed during the current study.
